# Immune Correlates of Disease Progression in Linked HIV-1 Infection

**DOI:** 10.3389/fimmu.2019.01062

**Published:** 2019-05-14

**Authors:** Michael Tuen, Jude S. Bimela, Andrew N. Banin, Shilei Ding, Gordon W. Harkins, Svenja Weiss, Vincenza Itri, Allison R. Durham, Stephen F. Porcella, Sonal Soni, Luzia Mayr, Josephine Meli, Judith N. Torimiro, Marcel Tongo, Xiaohong Wang, Xiang-Peng Kong, Arthur Nádas, Daniel E. Kaufmann, Zabrina L. Brumme, Aubin J. Nanfack, Thomas C. Quinn, Susan Zolla-Pazner, Andrew D. Redd, Andrés Finzi, Miroslaw K. Gorny, Phillipe N. Nyambi, Ralf Duerr

**Affiliations:** ^1^Department of Pathology, New York University School of Medicine, New York, NY, United States; ^2^Department of Biochemistry, University of Yaoundé 1, Yaoundé, Cameroon; ^3^Faculty of Medicine and Biomedical Sciences, University of Yaoundé 1, Yaoundé, Cameroon; ^4^Centre de Recherche du CHUM, Montréal, QC, Canada; ^5^Département de Microbiologie, Infectiologie et Immunologie, Université de Montréal, Montréal, QC, Canada; ^6^South African MRC Bioinformatics Unit, South African National Bioinformatics Institute, University of the Western Cape, Bellville, South Africa; ^7^Division of Infectious Diseases, Department of Medicine, Icahn School of Medicine at Mount Sinai, New York, NY, United States; ^8^Division of Intramural Research, National Institutes of Health-National Institute of Allergy and Infectious Diseases, Bethesda, MD, United States; ^9^Genomics Unit, Research Technologies Branch, Division of Intramural Research, Rocky Mountain Laboratories, NIAID, NIH, Hamilton, MT, United States; ^10^Medical Diagnostic Center, Yaoundé, Cameroon; ^11^Yaoundé General Hospital, Yaoundé, Cameroon; ^12^“Chantal Biya” International Reference Centre for Research on HIV/AIDS Prevention and Management, Yaoundé, Cameroon; ^13^Center of Research for Emerging and Re-Emerging Diseases, Institute of Medical Research and Study of Medicinal Plants, Yaoundé, Cameroon; ^14^School of Laboratory Medicine and Medical Sciences, Nelson R. Mandela School of Medicine, KwaZulu-Natal Research Innovation and Sequencing Platform, College of Health Sciences, University of KwaZulu-Natal, Durban, South Africa; ^15^Veterans Affairs New York Harbor Healthcare Systems, New York, NY, United States; ^16^Department of Biochemistry and Molecular Pharmacology, New York University School of Medicine, New York, NY, United States; ^17^New York University School of Medicine, Institute of Environmental Medicine, New York, NY, United States; ^18^Department of Medicine, Université de Montréal, Montréal, QC, Canada; ^19^Center for HIV/AIDS Vaccine Immunology and Immunogen Discovery, The Scripps Research Institute, La Jolla, CA, United States; ^20^Faculty of Health Sciences, Simon Fraser University, Burnaby, BC, Canada; ^21^British Columbia Centre for Excellence in HIV/AIDS, St. Paul's Hospital, Vancouver, BC, Canada; ^22^Department of Medicine, Johns Hopkins University, Baltimore, MD, United States; ^23^Department of Microbiology and Immunology, McGill University, Montréal, QC, Canada

**Keywords:** human immunodeficiency virus (HIV), epidemiologically-linked infection, BEAST, ADCC, IgA/IgG ratio, V1V2 antibody binding, viral signature K169, protective immune parameters and host factors

## Abstract

Genetic and immunologic analyses of epidemiologically-linked HIV transmission enable insights into the impact of immune responses on clinical outcomes. Human vaccine trials and animal studies of HIV-1 infection have suggested immune correlates of protection; however, their role in natural infection in terms of protection from disease progression is mostly unknown. Four HIV-1^+^ Cameroonian individuals, three of them epidemiologically-linked in a polygamous heterosexual relationship and one incidence-matched case, were studied over 15 years for heterologous and cross-neutralizing antibody responses, antibody binding, IgA/IgG levels, antibody-dependent cellular cytotoxicity (ADCC) against cells expressing wild-type or CD4-bound Env, viral evolution, Env epitopes, and host factors including HLA-I alleles. Despite viral infection with related strains, the members of the transmission cluster experienced contrasting clinical outcomes including cases of rapid progression and long-term non-progression in the absence of strongly protective HLA-I or CCR5Δ32 alleles. Slower progression and higher CD4/CD8 ratios were associated with enhanced IgG antibody binding to native Env and stronger V1V2 antibody binding responses in the presence of viruses with residue K169 in V2. ADCC against cells expressing Env in the CD4-bound conformation in combination with low Env-specific IgA/IgG ratios correlated with better clinical outcome. This data set highlights for the first time that V1V2-directed antibody responses and ADCC against cells expressing open, CD4-exposed Env, in the presence of low plasma IgA/IgG ratios, can correlate with clinical outcome in natural infection. These parameters are comparable to the major correlates of protection, identified *post-hoc* in the RV144 vaccine trial; thus, they may also modulate the rate of clinical progression once infected. The findings illustrate the potential of immune correlate analysis in natural infection to guide vaccine development.

## Introduction

In the quest for an effective HIV-1 vaccine, a definitive understanding of which immune responses should be induced to protect or control HIV infection remains elusive. Passive immunization experiments with broadly neutralizing antibodies (bnAbs) in animal models of HIV-1 infection have succeeded in blocking or halting infection (1, 2). However, it has not been possible to elicit such HIV-1 Abs through active vaccination (3). The only vaccine study that showed modest protection from HIV-1 infection of 31.2% after 3.5 years (modified intention-to-treat analysis), RV144, suggested that non-neutralizing Abs (nnAbs) directed against the V1V2 envelope region inversely correlated with infection risk (4–6). Sieve analysis of breakthrough viruses in RV144 revealed imprints of Ab selection pressure at residues K169 and I181 in the V2 envelope region (7). Furthermore, high levels of antibody-dependent cellular cytotoxicity (ADCC) in association with low plasma levels of Env-specific IgA Abs or IgA/IgG ratios were identified as correlates of protection (4, 8). Immune correlates of protection, as suggested after RV144, have been replicated partly in non-human primates (9, 10); however, subtle differences exist between humans and monkeys with regards to immune responses and antibody repertoires, such as the structure and functionality of IgG3 and IgA subclasses (11, 12). Studies of natural HIV-1 infection enable the analysis of protective immune patterns in the human system; however, it is not yet clear to what extent correlates of disease progression mirror correlates of protection from infection. A better understanding of protective immune responses in natural infection and how this knowledge can be used to direct vaccine research is needed.

Overall, protection from infection in the context of vaccination has been primarily linked to humoral immune responses, whereas the better clinical outcome of natural infection has been attributed mainly to host factors such as protective HLA alleles and associated cellular responses (13, 14). Recent research suggests that polyfunctional immune responses, including IgG1- and/or IgG3-based Fc-mediated antibody functions, may be pivotal for protective effects and better clinical outcome (15–17). Immune correlates of protection, as identified *post-hoc* in RV144 have not yet been confirmed in natural infection. Despite numerous publications confirming the immune pressure exerted by the RV144 vaccine regimen and the immunologic and viral evidence of protection, the findings from RV144 remain controversial.

Here we describe a multifactorial analysis of immune, viral, host, and clinical parameters studied for 15 years in four HIV^+^ Cameroonian adults, including a polygamous male transmitting HIV to two females. Immune responses in these HIV^+^ individuals were compared to the clinical outcomes. The results of these analyses provide insights into the impact and plasticity of protective immune parameters after infection with related viral strains.

## Results

### Transmission Events and Clinical Outcomes in Individuals Infected With Related HIV-1 Strains

**Figure 1:**
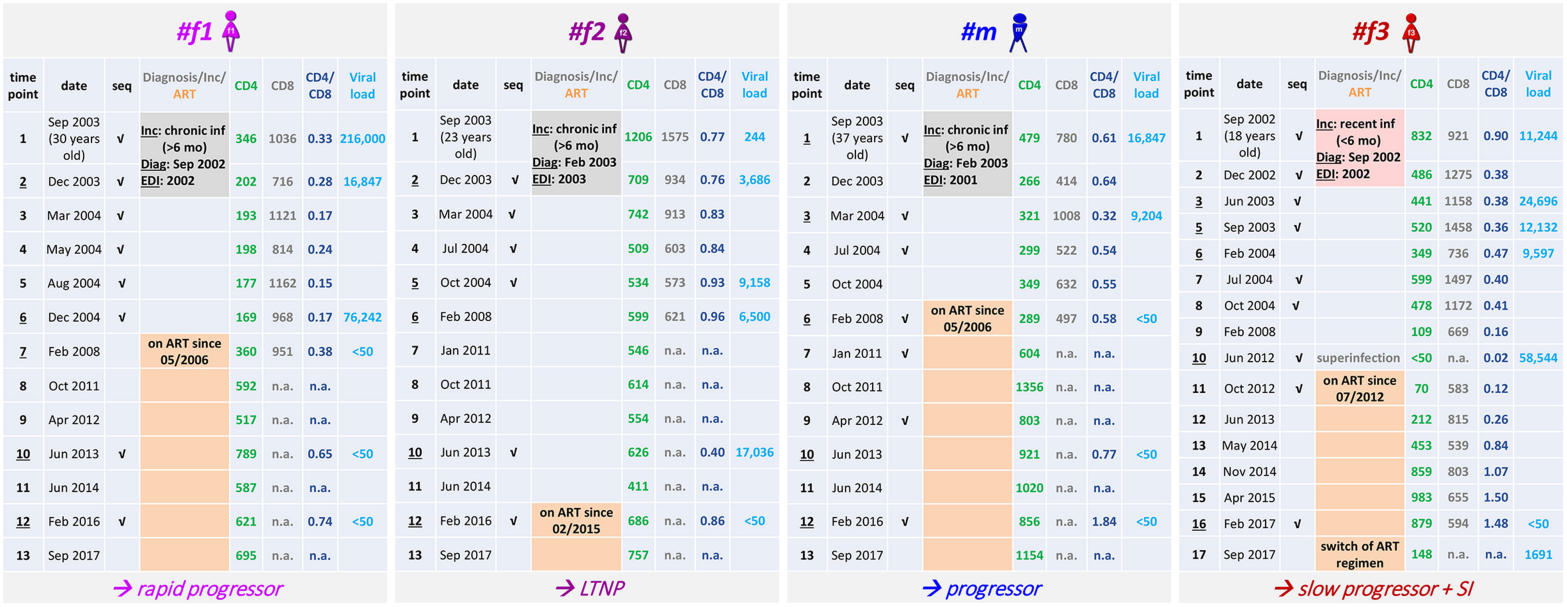
Clinical parameters of the four Cameroonian HIV+ study participants. Table summarizing longitudinal study time points, dates of sampling, the age of participants, accomplished viral sequencing (seq) and selected clinical parameters. The results of incidence testing (Inc) are shaded in gray (#f1, #f2, #m) or pale red (#f3) according to sampling start at a stage of chronic infection (>6 months) or recent infection (<6 months), respectively. In addition, the dates of diagnosis (Diag) and the estimated dates of infection (EDI) based on BEAST analyses (Figure 2) are indicated. The time point when superinfection (SI) was detected in #f3 is labeled (gray). Time points at which the participants received antiretroviral treatment (ART) are highlighted in orange with the ART start date indicated. The clinical classification as long-term non-progressor (LTNP, #f2) is based on CD4 counts >500 cells/μL in the absence of AIDS-defining symptoms for >8 years without ART. Slow progression (#f3) is defined as the absence of AIDS-defining symptoms without ART for >8 years. The classification as progressor (#m) is based on CD4 counts dropping below 300 cells/μL and ART initiation within 4 years after diagnosis; rapid progression (#f1) is defined by CD4 counts repeatedly dropping below 200 cells/μL within 4 years after diagnosis and estimated date of infection.

**Figure 2:**
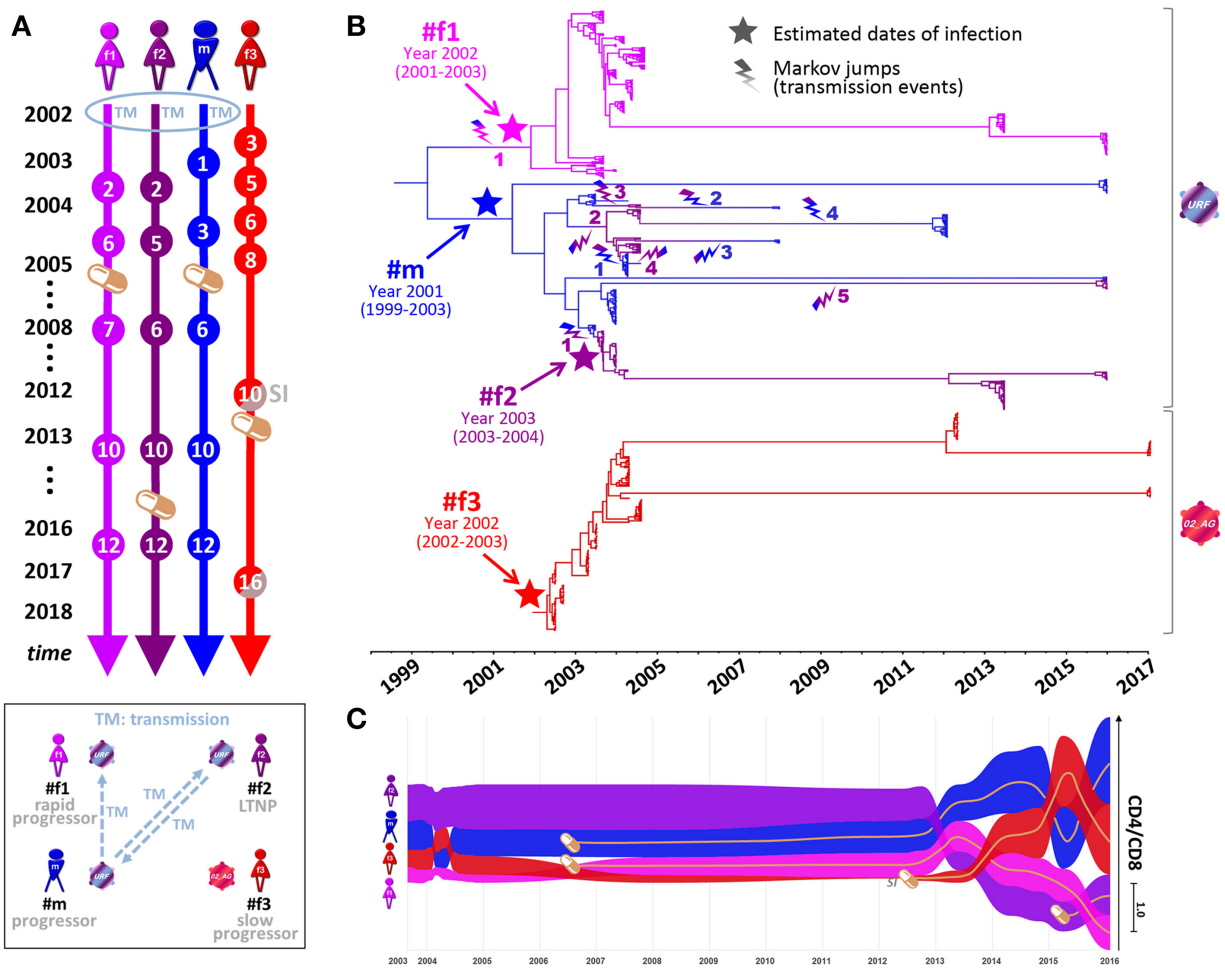
Phylogenetic analysis, transmission events, and clinical parameters in four Cameroonian HIV^+^ individuals. **(A)** Timeline of essential study time points (spheres). Orange pill icons indicate time points of antiretroviral treatment initiation. TM and SI indicate the occurrence of transmission or superinfection events, respectively. **(B)** Temporal estimation of epidemiologically-linked transmission events and dates of infection using BEAST time-calibrated MCC trees (564 functional *env* sequences, 2002–2017, HIV region 6225–7817 according to HXB2 numbering). The branches of the trees are color-coded according to each individual. A color gradient along the branches and a lightning symbol (same color gradient) indicate historical transmission events among individuals. Transmission events are numbered consecutively for each recipient. The median estimated dates of infection (including 95% highest posterior density) are labeled and highlighted in the tree with a star. **(C)** Bump chart illustrating longitudinal CD4/CD8 ratios in the four study participants along the timeline on the X-axis modeled based on available data points. The magnitude of CD4/CD8 ratios is indicated on the Y-axis, sorted in descending order. Pill icons and brown lines indicate long-term antiretroviral treatment. BEAST, Bayesian evolutionary analysis by sampling trees; LTNP, long-term non-progressor; MCC, maximum clade credibility; URF, unique recombinant form. Inset, Schematic overview of transmission events and clinical classifications (2002–2017) of the four participants. Virus symbols indicate genetically related strains of subtypes URF (blue-purple) and CRF02_AG (red).

**Figure 3:**
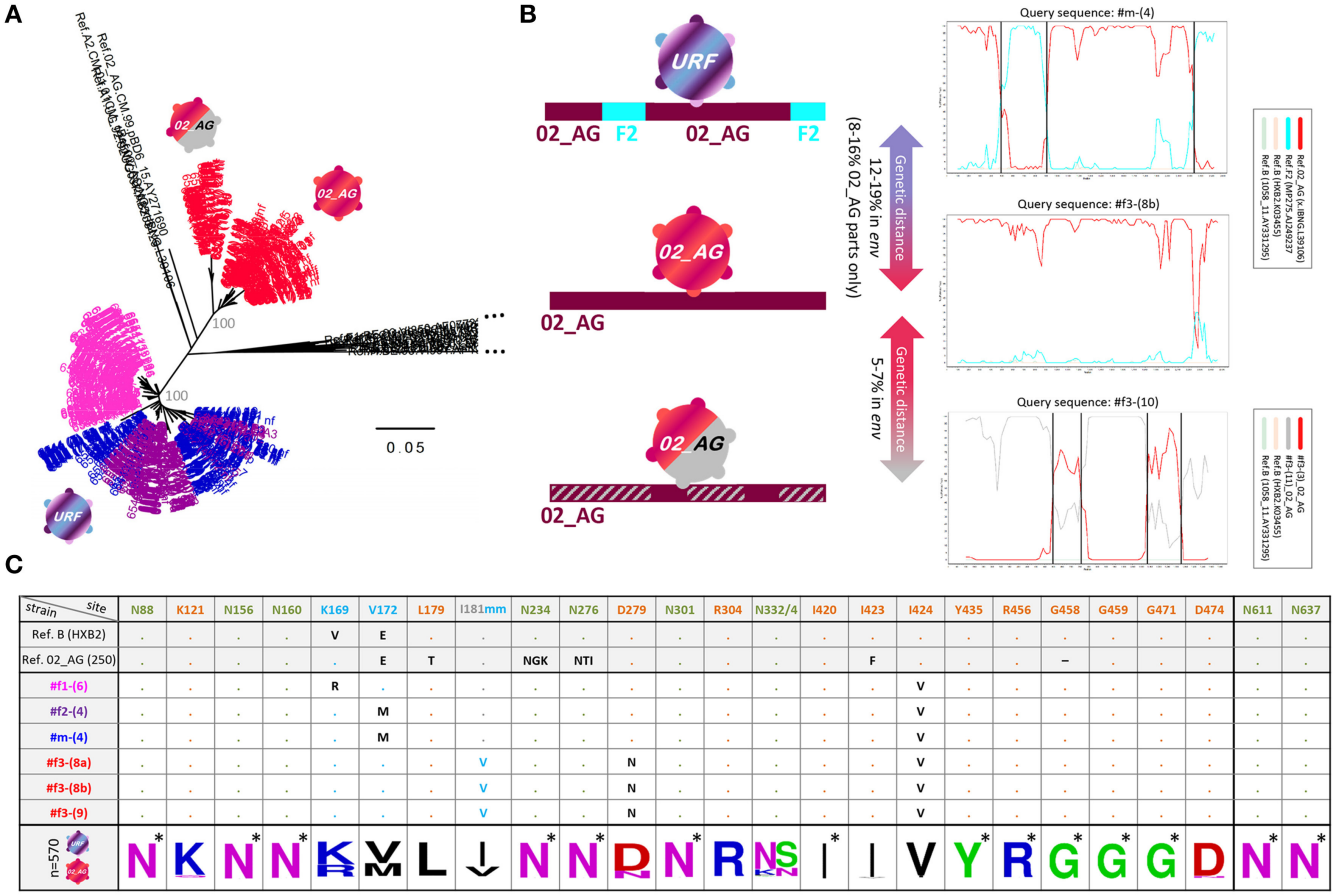
*Env* genetic analysis in four Cameroonian HIV^+^ individuals. **(A)** Neighbor-joining phylogenetic trees (Kimura two-parameter model) displaying *env* sequences (HIV region 6225-7817 according to HXB2 numbering). Taxa are color-coded according to the participant (Figure 2). For each study participant, all sequences from multiple time points are displayed, i.e., ≥20 *env* sequences per time point (Figure 1). Bootstrap values are indicated in gray for the major branches of the participants' CRF02_AG and unique recombinant form (URF) sequences. The bar indicates a genetic distance of 5%. Reference sequences (LANL Database) are shown in black. CRF02_AG sequences of participant #f3 are separated into two populations: pre-superinfection (red icon; time points 1-9) and post superinfection (red-gray icon; time points ≥10). **(B)** Schematic representation of *env* recombination patterns in the three viral populations of the four study participants (left) as determined in Simplot bootscan analyses (right). The horizontal bar indicates the entire *env* genomic region; F2 parts are displayed in turquoise, CRF02_AG parts are displayed in dark red, and dashed gray indicates sequences of a genetically distant (>5%) CRF02_AG variant. Simplot Bootscan analyses were performed using representative query sequences for each virus population (indicated on top of each plot) against subtype CRF02_AG (red), F2 (turquoise) and B (light brown and green, outlier) reference sequences (boxed). The window width, step size, and bootstrap replicates were set to 200 bp, 20 bp, and 100, respectively. The Y-axis indicates bootstrap support; the X-axis indicates the *env* region. Recurring breakpoints supported by bootstrap values >70% are indicated as vertical black lines. The full range of genetic distances in *env* between viral populations is indicated (middle). **(C)** Env epitope analysis of six representative single genome amplified (SGA) sequences (middle), isolated from the four HIV+ individuals. At the bottom, a sequence logo analysis is shown for the entire set of studied Env sequences; asterisks indicate 100% conserved sites. Reference sequences of clade B (HXB2) and CRF02_AG (250) are shown on top. N-glycosylation sites critical for broadly neutralizing antibodies (bnAbs) are colored in olive green: N88 (gp120/gp41 interphase bnAb 35O22), N156 and N160 (V2 glycan bnAbs, e.g., PG9/PG16), N234 and N276 (gp120/gp41 interphase bnAb 8ANC195), N301 and N332/N334 (V3 glycan bnAbs, e.g., PGT121/PGT128) and N611 and N637 (gp120/gp41 interphase bnAb PGT151). Sites of immune pressure in the RV144 vaccine trial (K169, V172, and I181 mm (mismatch) in V2) are highlighted in light blue. Possible sites of resistance to CD4 binding site bnAbs are highlighted in orange. Dots indicate the presence of the above-listed amino acid residue. Black entries (or light blue in the case of I181 mm) indicate divergence from the above-listed amino acid.

Four Cameroonian HIV^+^ individuals, one male (#m) and three females (#f1, #f2, and #f3), were studied longitudinally from 2002 to 2017 (Figures 1, 2). #m and #f1 experienced a progressive course of the disease, whereas #f2 and #f3 were a long-term non-progressor (LTNP) and a slow progressor, respectively (Figures 1, 2C. Epidemiologic linkages among participants #m, #f1, and #f2 were confirmed through their genetically related unique recombinant form (URF) viruses (the mosaic composition of subtypes CRF02_AG and F2), which comprised a monophyletic clade with high statistical support in *env* phylogenetic trees (Figures 2B, 3A; Supplementary Figures 1, 2, and Supplementary Table 1). To trace the history of the infecting strains among the partners in the polygamous heterosexual relationship, we performed Bayesian evolutionary analyses by sampling trees (BEAST), summarized in the form of a time-calibrated tree (Figure 2B and Supplementary Figure 1). Two distinct phylogeny–phenotypic trait-correlation patterns were consistently recovered. First, #f1 virus sequences formed a monophyletic sub-clade within the larger URF clade. This would be expected if the initial infection of #f1 involved the transmission of a single strain, with the most probable donor being #m during 2002. Second, #f2 and #m URF sequences were highly interspersed within a single clade on the tree, suggesting numerous transmissions in both directions, most likely commencing with the transmission of a URF virus from #m to #f2 in 2003 (Figures 1, 2B, Supplementary Figure 1, and Supplementary Table 1). Not surprisingly, inter-host transmissions most frequently involved #m, either as a recipient or donor, reflecting the central role this participant has likely played in the epidemiology of the three linked individuals, and consistent with the linked participants' self-reported sexual activity (see Methods).

Participant #f3 became infected with a CRF02_AG strain, which is phylogenetically different from the URF strains but harbors genetic similarities with the mosaic URF sequence through matching subtype CRF02_AG parts (Figure 3B). Longitudinal follow-up showed that at later time points (starting at time point ten), #f3 exhibits genetically distant variants >5% in *env* (5.2–7.1%) (Figures 3A,B and Supplementary Figure 2), coincident with an increase in viral load and drop in CD4 levels and CD4/CD8 ratios (Figures 1, 2C). This suggests the occurrence of intra-subtype CRF02_AG superinfection from an external source between time points nine and ten. Env breakpoint analysis revealed the occurrence and outgrowth of secondary recombinants between the superinfecting and the preexisting CRF02_AG variants (Figure 3B and Supplementary Figure 2).

### Differential Neutralization Responses and Cell-Surface Env Binding

**Figure 4:**
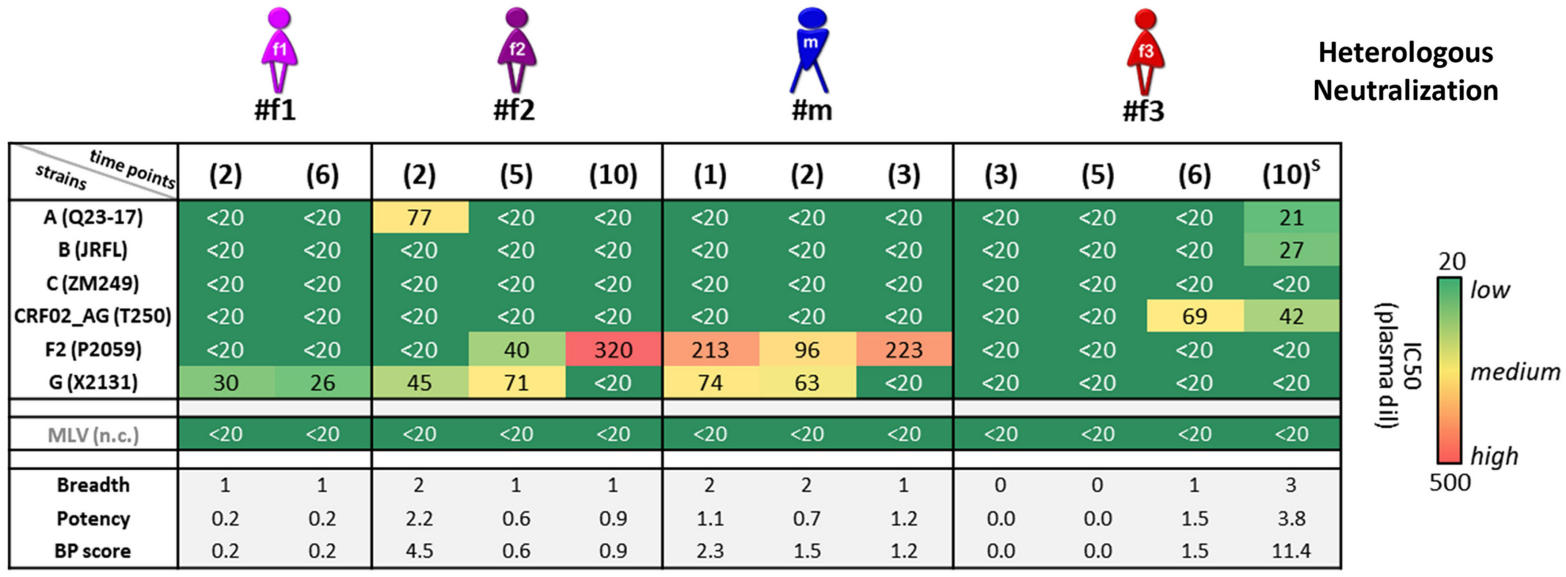
Heterologous neutralization in study participants. Heterologous neutralization responses of longitudinal plasma samples (time points in brackets; S: detected superinfection) against six reference pseudoviruses (Env subtypes and viral strains listed); murine leukemia virus (MLV) was used as negative control (n.c.). Mean plasma dilutions (dil) needed for 50% neutralization (IC_50_) were calculated from two independent experiments in duplicates and are illustrated in a heat map. Breadth, potency, and combined breadth-potency (BP) scores were calculated for each sample separately and in relation to mean IC_50_ values of the studied pseudoviruses.

Heterologous neutralization was studied using longitudinal plasma samples from time points before the participants started antiretroviral treatment (ART), and a set of reference HIV-1 viruses was selected with consideration of the clades of the participants' infecting viruses (Figure 4 and Supplementary Figure 3). The heterologous neutralization response in URF-infected participants #f1, #f2, and #m remained narrow, principally directed against clade F2 and G pseudoviruses, with higher potencies in #f2 and #m compared with #f1. CRF02_AG-infected participant #f3, who entered the study within 6 months post-infection, had undetectable IC_50_ values at the initial study time points, yet developed a CRF02_AG-focused neutralization response at time point six and greatest breadth after superinfection at time point 10 with three of six pseudoviruses neutralized (Figure 4 and Supplementary Figure 3). Env epitope analysis revealed highly conserved neutralizing epitopes across longitudinal viral sequences of the four participants at key sites of vulnerability on the gp120 and gp41 Env trimer. Greater variation was observed in non-neutralizing (nn) epitopes of V2, linked to immune pressure in the RV144 vaccine trial (Figure 3C).

Neutralization experiments using autologous pseudoviruses, encoding URF and CRF02_AG *envs*, revealed a more balanced neutralization pattern among participants (Supplementary Figure 4). Notably, compared with URF, the CRF02_AG pseudovirus appeared significantly more sensitive to neutralization (IC_50_ values and maximum neutralization), mirrored by greater binding of plasma and weakly-neutralizing or nnAbs to the surface of native Env-expressing cells. In particular, the CRF02_AG Env was more accessible to Abs targeting V2 (CH59) and V3 (19b) (Supplementary Figure 4).

### Contrasting Envelope-Specific IgA/IgG Ratios and ADCC (N-U-) Levels

**Figure 5:**
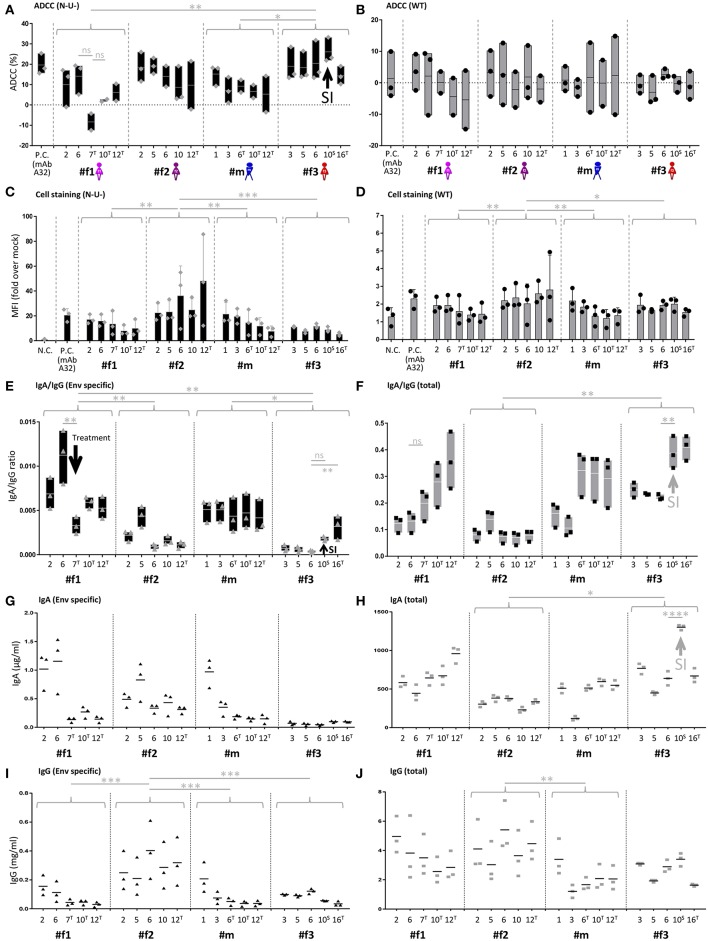
IgA/IgG levels, cell-associated Env binding and antibody-dependent cellular cytotoxicity (ADCC). **(A,B)** Longitudinal ADCC analysis using Nef- and Vpu-deficient (N-U-) **(A)** and wild-type (WT) **(B)** HIV-1 ADA infected CEM-NKR cells in plasma samples of the four study participants. **(C,D)** Longitudinal analysis of cell-associated Env binding (cell staining) using HIV-1 ADA infected CEM-NKR cells. Infection experiments were performed both with N-U- **(C)** and WT virus **(D)**. **(E,F)** Longitudinal analysis of Env-specific **(E)** and total **(F)** IgA/IgG ratios. **(G,H)** Longitudinal analysis of Env-specific **(G)** and total **(H)** IgA levels. **(I,J)** Longitudinal analysis of Env-specific **(I)** and total **(J)** IgG levels. On the X-axis, participant IDs are listed chronologically by time point. T and S in superscript indicate time points where participants were on antiretroviral treatment or at superinfection (SI), respectively. Gray arrows indicate selected patterns of changes upon superinfection or initiation of antiretroviral treatment. MAb A32 was used as a positive control (P.C.) and mock-infected cells and/or HIV negative plasma as a negative control (N.C.). Means of two or three independent experiments performed in duplicates (IgA and IgG quantitations) or triplicates (ADCC and cell stainings) are shown. Also, floating bars (min to max) are shown in **(A,B,E,F)** and standard deviations in **(C,D)**. All statistically significant differences between study participants are indicated as well as selected statistical comparisons between two-time points (One-way ANOVA, ^*^P < 0.05; ^**^P < 0.005; ^***^P < 0.0005; ^****^P < 0.0001; ns, not significant).

**Figure 6:**
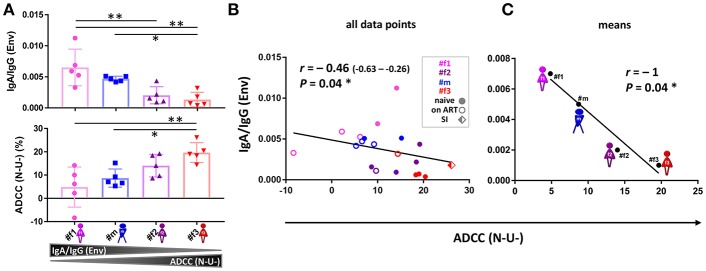
ADCC and IgA/IgG patterns. **(A)** Scatter plot analysis of Env-specific plasma IgA/IgG ratios (top) and ADCC (N-U-)(bottom) against HIV-1 ADA-infected CEM-NKR cells. Mean and standard deviation for the five longitudinal values per participant are shown. All statistically significant differences among participants are indicated (one-way ANOVA, ^*^P < 0.05; ^**^P < 0.005). **(B,C)** Correlation analysis between ADCC (N-U-) and Env-specific plasma IgA/IgG ratios using all data points **(B)** and means per participant and parameter **(C)**. Correlation coefficients *r* (with 95% confidence intervals if applicable) and *P*-values are indicated using non-parametric Spearman rank. Asterisks indicate statistically significant correlations in a two-tailed **(B)** or one-tailed Spearman rank test (means) **(C)** (^*^P < 0.05). ADCC, antibody-dependent cellular cytotoxicity; ART, antiretroviral treatment; N-U-, Nef- and Vpu-deficient.

Longitudinal samples were studied for IgA, IgG, and ADCC levels (Figures 5, 6 and Supplementary Figures 5, 6). ADCC was determined in infection experiments with wild-type (WT) or Nef- and Vpu-deficient (N-U-) virus, the latter exposing Env in the CD4-bound conformation at the cell surface (18). Deletion of Vpu also results in an accumulation of tethered viral particles at the cell surface, mediated by the restriction factor BST-2 (19), thus resulting in an accumulation of Env at the cell surface (18, 20). Beside downregulating CD4, Nef also downregulates NKG2D ligands, a mechanism also known to decrease ADCC responses (21, 22). The N-U- virus was used to amplify ADCC responses, since it has been reported that ADCC-mediating Abs present in HIV+ sera preferentially target the open CD4-bound Env conformation (23). ADCC (WT) was generally low and at comparable levels, as reported in cohorts of HIV-1 infected individuals (23). ADCC (N-U-) was substantially higher in most samples, and we observed the highest ADCC (N-U-) and lowest Env-specific IgA/IgG ratios in the LTNP (#f2) and the slow progressor (#f3) (Figures 5A,B,E, 6). This association was Env-specific since total IgA/IgG ratios showed a different pattern (Figure 5F). The low Env-specific IgA/IgG ratios were primarily attributed to low IgA levels in #f3 but to high IgG levels in #f2. Superinfection in #f3 markedly increased total IgA and IgA/IgG ratios and slightly elevated Env-specific IgA/IgG and ADCC (N-U-) levels (Figures 5A,E,F,H). Using individual data points or means per patient and parameter, we found an inverse correlation of Env-specific IgA/IgG ratios with ADCC (N-U-) but not with ADCC (WT) (Figures 5B,C and Supplementary Figure 6).

### Divergent Env-Directed Binding Responses in Slow-Progressing Individuals

**Figure 7:**
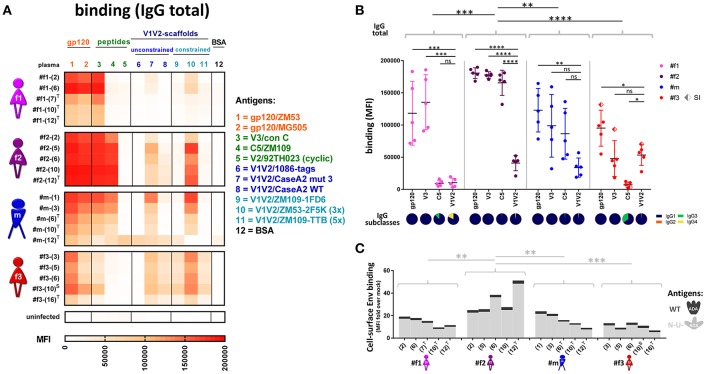
Antibody binding profiles of study participants. **(A)** Heatmap of longitudinal total IgG antibody binding levels, determined by a multiplex bead assay (Luminex) using plasma diluted 1:200 against a set of eleven Env antigens. Antigen #10 is trimeric (3x), and #11 is pentameric (5x). BSA coated beads and plasma from an uninfected, healthy Cameroonian individual were used as negative controls. ^T^: antiretroviral treatment; ^S^: superinfection. **(B)** Quantitative scatter plot analysis of total IgG levels against gp120, V3, C5, and V1V2. For gp120 and V1V2, the average binding levels of two gp120 proteins or six scaffolded V1V2 proteins (see **A**) are shown, respectively. The dots represent the different time points per participant for the corresponding antigen; SI: superinfection. All statistically significant differences (one-way ANOVA) in overall binding levels among individuals are indicated, as well as statistical analyses comparing the binding of every antigen against V1V2 within each individual. IgG subclass composition (IgG1-4) of plasma binding to the four selected Env antigens (averaged longitudinal values) are shown as pie charts according to the indicated color-code. **(C)** Cell-surface staining of CEM-NKR cells infected with a virus encoding ADA wild-type (WT, dark gray, top) or Nef-, Vpu-deficient (N-U-, light gray, bottom) virus. Staining of native Env was done using longitudinal plasma samples (time points in brackets) and is displayed in stacked bar graphs with means of at least two repetitive experiments. Statistical analysis was performed for the N-U- data set using one-way ANOVA. ^*^P < 0.05; ^**^P < 0.005; ^***^P < 0.0005; ^****^P < 0.0001; ns, non-significant.

**Figure 8:**
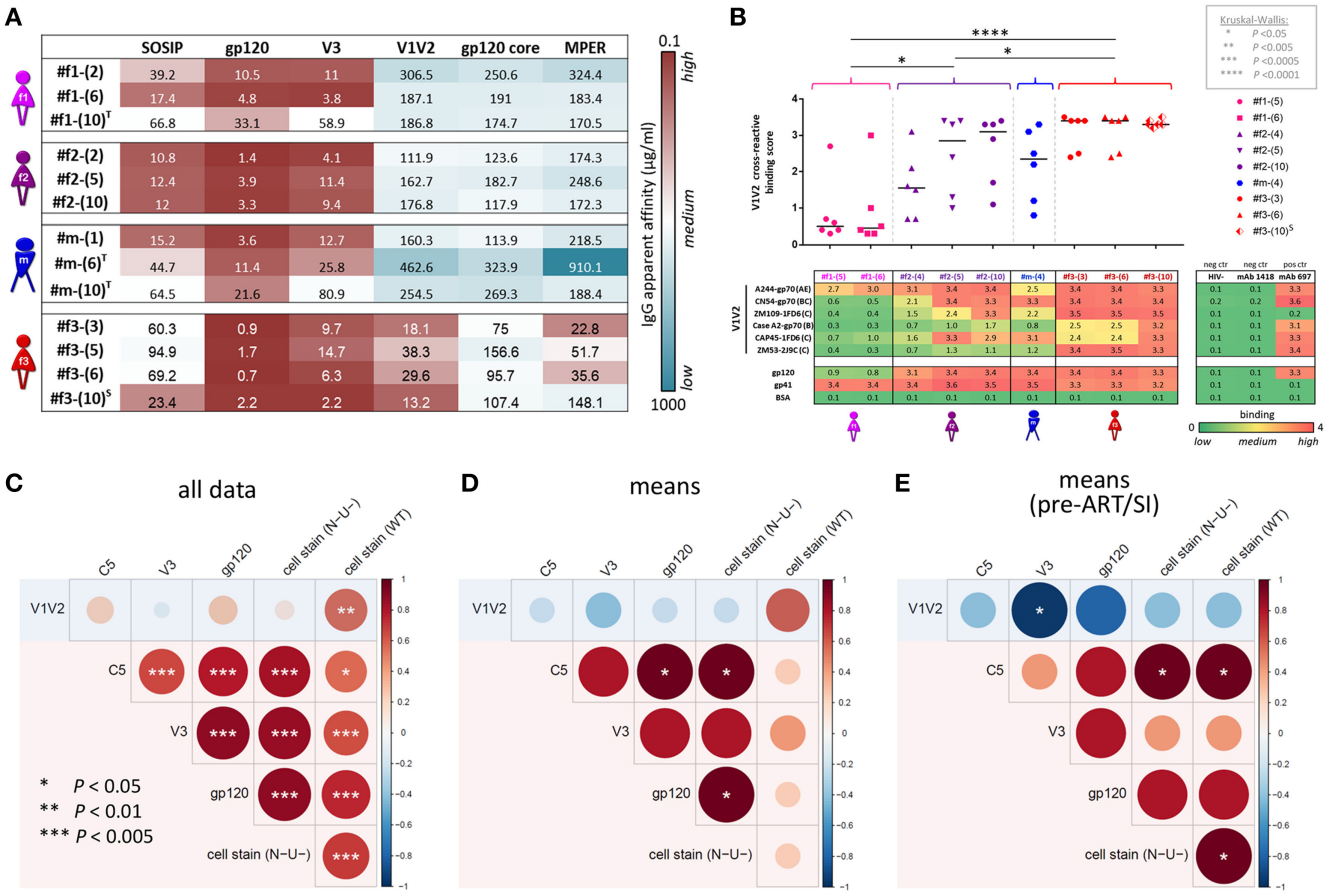
IgG apparent affinities, ELISA plasma binding levels, and binding correlation analysis. **(A)** Heatmap of half-maximum binding concentrations (EC50, μg/mL) determined by ELISA using plasma-purified IgG (range of 0.2–500 μg/mL) from longitudinal time points (shown in parentheses). A set of six Env antigens was used: trimeric SOSIP/BG505, gp120/JRFL, cyclic V3/ZM109 peptide, constrained V1V2/ZM109-1FD6 scaffold, gp120 core/JRFL, and MPER gp41/con B peptide. T and S in superscript indicate time points where participants were on antiretroviral treatment or at superinfection, respectively. **(B)** Plasma samples diluted 1:100 were tested against six different V1V2 constructs: V1V2-gp70 fusion proteins A244 (clade AE), CN54 (clade B/C), and CaseA2 (clade B), V1V2-1FD6 fusion proteins ZM109 and CAP45 (both clade C), as well as trimeric V1V2-2J9C fusion protein ZM53 (clade **C**). Antigens gp120 MN (clade **B**) and gp41 MN (clade **B**), as well as anti-V2 mAb 697-D, were used as positive controls; BSA, plasma of a healthy HIV^−^ Cameroonian individual and anti-parvovirus B19 mAb 1418 were used as negative controls. A heat map of binding levels is shown at the bottom. The V1V2 binding responses are summarized for each time point sample to a V1V2 cross-reactive binding score, shown on top. V1V2 cross-reactive binding scores were statistically compared between participants using one-way ANOVA; asterisks indicate statistically significant differences according to the boxed *P*-value scheme. Bi-colored diamonds as symbols indicate the time point when superinfection (S in superscript) was detected in #f3. **(C–E)** Correlation analysis between Luminex V1V2, V3, C5, gp120, and cell-associated Env binding levels. Linear regression analysis was performed for the following data sets: all individual data points of the four participants **(C)**, binding means per participant and antigen **(D)** and means per participant/antigen from time points pre-ART and pre-superinfection **(E)**. Correlograms are shown, sized and color-coded according to the correlation coefficient (*r*). Asterisks indicate all correlations that reached statistical significance in a two-tailed Spearman rank test **(C)** or one-tailed Spearman rank test for the means **(D,E)** according to the provided *P*-value scheme (on the left). Red background indicates the presence of a positive correlation pattern; the blue background indicates the tendency of an overall inverse or absence of correlation pattern. ART, antiretroviral treatment.

Comprehensive binding analyses were performed using multiplex bead-based Luminex experiments, cell-surface Env staining, and enzyme-linked immunosorbent assays (ELISA) (Figures 7, 8). Multiplex binding analyses against eleven different Env antigens (Figures 7A,B) and cell-based staining of heterologous (clade B ADA) **Env** (Figures 5C,D, 7C) revealed IgG1-dominated binding responses, with greatest binding levels observed in LTNP #f2. Overall binding levels were lowest in slow progressor #f3. However, she exhibited the highest mean V1V2-directed responses (Figure 7B), mirrored in strong apparent affinities of plasma-purified IgG and ELISA plasma binding against six different V1V2 fusion proteins (Figures 8A,B). Within the transmission cluster, the binding patterns and strengths varied substantially despite infection with related viruses (Figures 7, 8). While binding patterns were qualitatively comparable in #f2 and #m, binding levels were far weaker in #m compared to #f2 (Figures 7A,B). In contrast, #f1, whose viral population evolved more independently after the initial transmission event compared to the co-evolving participants #m and #f2, also exhibited a strikingly different binding pattern (Figures 7A,B). Specifically, in #f1, V3 binding levels were relatively the strongest and C5 binding seemed abrogated. These differential binding patterns were mirrored by strong variations at 10 positions in #f1's viral V3 sequences compared to #m and #f2 who evolved viruses with highly similar V3 sequences. Within C5, only minor variations at one position were observed in #f1's viruses compared to both #m and #f2 (Supplementary Figure 7).

### V1V2 Antibody Binding Responses and Viral Sequence Patterns

**Figure 9:**
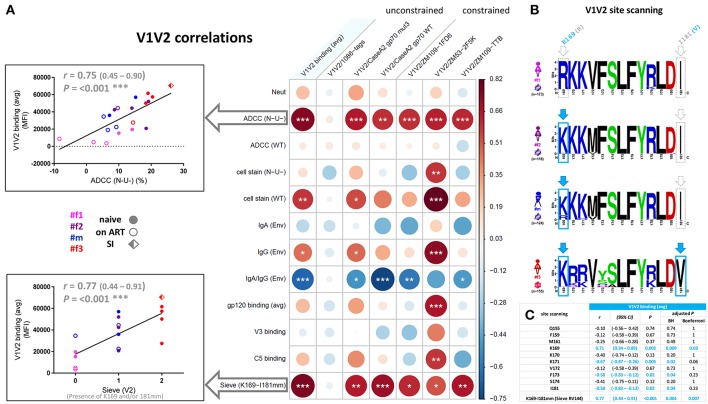
Correlation analysis of the V1V2 binding response and epitope site-scanning. **(A)** Correlation analysis of V1V2 binding levels with selected immune responses and the presence of residue K169 and mismatch (mm) at I181 in contemporaneous viral sequences (according to the sieve analysis in the RV144 vaccine trial). In the correlogram, circles are sized and color-coded according to linear regression coefficients (*r*) between the indicated parameters. Asterisks indicate all statistically significant correlations (Spearman rank, ^*^P<0.05; ^**^P<0.01; ^***^P<0.005). The two most significant correlations with V1V2 binding (avg) are depicted as scatter plots on the left (95% confidence intervals in brackets). Sieve (V2) on the x-axis is scaled according to the presence of K169 (score of 1), a mismatch at I181 (score of 1), or both (score of 2) in contemporaneous viruses of participants. ART: antiretroviral treatment; avg: average; Env, Env-specific; naïve, ART-naïve; SI, superinfection; WT, wild-type infection; N-U-, Nef- and Vpu-deficient virus infection. **(B)** Sequence logo analysis of the immunodominant V1V2 region (Env amino acid region 169-181 according to HXB2 numbering) from all functional viral sequences of the four study participants. The presence of residue K169 or mismatch of I181 is boxed and highlighted in light blue. **(C)** Site-scanning analysis of all non-conserved amino acid sites within the immunodominant V1V2 region of longitudinal viral sequences from the four study participants. Spearman rank correlations were performed between longitudinal V1V2 binding (avg) responses and the presence of the indicated amino acid residue(s) in the contemporaneous viruses. Correlation coefficients *r*, 95% confidence intervals (CI), significance *P*, and multiple-comparisons-adjusted *P*-values (Benjamini Hochberg and Bonferroni method) are displayed for each correlation; statistically significant results (*P* < 0.05) are highlighted in light blue.

Correlation analyses revealed that binding of gp120, V3, and C5 positively correlated with one another but not with V1V2. In contrast, V1V2 binding was mostly an independent variable, which did not consistently correlate with any other binding response except the inverse correlation with V3 binding for time points pre-ART and pre-superinfection (Figures 8C–E). V1V2 binding did not correlate with ADCC (WT), however, V1V2 binding significantly correlated with ADCC (N-U-) and a viral sequence signature in V2 (presence of K169 and a mismatch of I181) of the contemporaneous viruses in the participants (Figure 9A). This viral sequence pattern had also been associated with increased vaccine efficacy in RV144 (7). Significant correlations were still maintained among the three linked individuals exclusively (Supplementary Figure 8). Based on these findings, we performed a site-scanning correlation analysis of all non-conserved amino acid sites within the immunodominant V1V2 region (Figures 9B,C and Supplementary Figure 9). The strongest correlation with V1V2 binding was found for K169 (Figure 9C), which remained significant after Benjamini-Hochberg (BH) multiplicity correction and using data exclusively from the three epidemiologically-linked individuals (Supplementary Figure 9). K169 was absent in #f1 but was present in most viral sequences of #f2 and #f3 (Figure 9B; Supplementary Figure 9, and Supplementary Table 2). Notably, the presence of residue K169 correlated positively with ADCC (N-U-) and inversely with Env-specific IgA/IgG ratios (Supplementary Figure 9).

### Viral and Immunologic Factors and Clinical Outcome in the Four Related HIV^+^ Individuals

**Figure 10:**
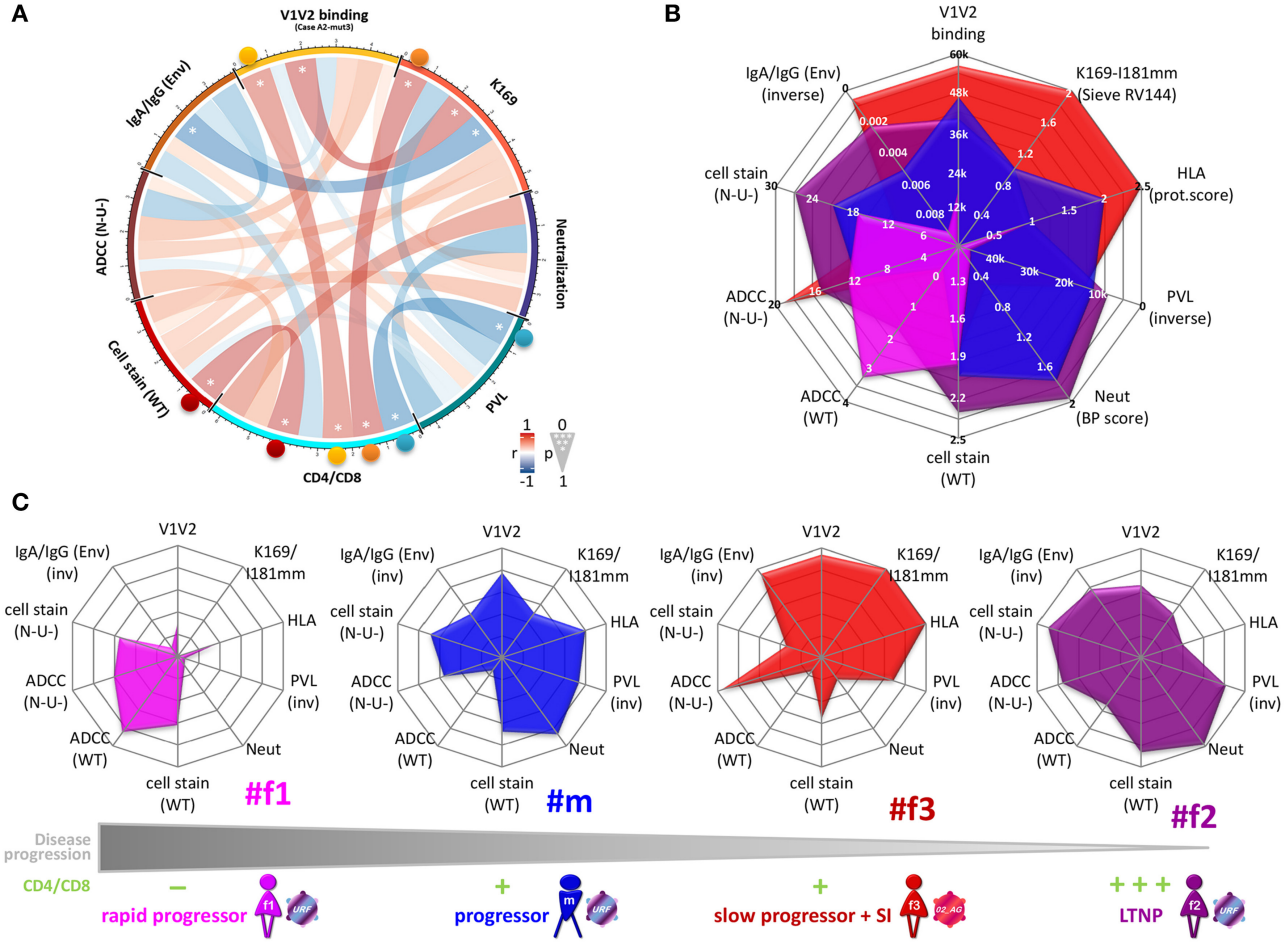
Clinical correlation analysis with viral, immunologic, and host parameters. **(A)** Chord diagram illustrating the network of linear correlations among eight major viral, immunologic, and clinical factors. Chords are color-coded according to the magnitude of the correlation coefficient (*r*); chord width inversely corresponds to the *P*-value. Two-tailed Spearman rank tests were performed using data points at time points pre-ART and pre-superinfection. Asterisks indicate all statistically significant correlations within chords (^*^P < 0.05). Colored spheres highlight statistically significant linear correlations with CD4/CD8. **(B,C)** Radar plots comparing the inter-individual patterns of ten selected viral, immunologic, host, and clinical factors (means from data points pre-ART and pre-superinfection). **(B)** Overlay of radar plots from the four participants. Scaling of the radial axes is indicated and the direction goes from low protective values in the center to high protective values at the rim of the octagons (inv: inverse order). **(C)** Individual radar plots are ordered from left to right, according to lower disease progression and higher CD4/CD8 ratios. Mean CD4/CD8 ratios from time points pre-ART and pre-superinfection are classified as: 0–0.25 (–); >0.25–0.5 (+); >0.5–0.75 (++); >0.75 (+ + +). Env, Env-specific; HLA, protective HLA scoring; WT, wild-type infection; Neut, Neutralization breadth-potency score; N-U-, Nef- and Vpu-deficient virus infection; PVL, plasma viral load; V1V2, averaged binding to scaffolded V1V2 antigens.

To screen for parameters associated with clinical outcome, we performed correlation analyses, selectively using time points pre-ART and pre-superinfection (Figure 10A; Supplementary Figure 10, and Supplementary Table 2). A few individual parameters correlated with CD4/CD8 ratios including Env-specific IgG levels and cell-surface staining of native Env (BH method). None of these correlations remained significant when tested exclusively among the three epidemiologically-linked participants with multiplicity correction (Supplementary Figure 10). However, Ab responses directed against the antigen V1V2/CaseA2-mut3 (carrying K169)(24), in the presence of viruses with K169 strongly correlated with CD4/CD8 ratios. Furthermore, ADCC (N-U-) in combination with Env-specific IgA/IgG levels, yielded significant multivariate correlation with CD4/CD8 (Table 1 and Supplementary Figure 10). ADCC (WT) together with Env-specific IgA/IgG ratios did not correlate with CD4/CD8 ratios among the four participants. Screening for the presence of protective host or viral factors, we identified no CCR5Δ32 mutant genotypes and no between-host differences in viral *nef* genes. However, we identified a protective HLA-B^*^57 allele with an adaptive viral *gag* mutation (T242N) in the slow progressor #f3 (Table 2).

**Table 1:** Clinical correlation analysis with viral and immunologic parameters.

			**CD4/CD8**		
				**Adjusted P**	
	***r***	**(95% CI)**	***P***	**BH**	**Bonferroni**
**SINGLE PARAMETER**					
Neut	**0.66**	**(0.20 – 0.88)**	**0.05**	0.12	0.45
ADCC (N-U-)	0.40	(0.13 – 0.62)	0.22	0.28	0.90
ADCC (WT)	−0.01	(−0.02 – 0)	0.97	0.97	1.00
cell stain (N-U-)	**0.64**	**(0.23 – 0.86)**	**0.04**	0.07	0.34
cell stain (WT)	**0.70**	**(0.26 – 0.90)**	**0.02**	**0.05**	0.24
IgA (Env)	−0.23	(−0.38 – −0.07)	0.50	0.54	1.00
IgG (Env)	**0.72**	**(0.27 – 0.91)**	**0.02**	**0.05**	0.21
IgA/IgG (Env)	−0.37	(−0.58 – −0.12)	0.26	0.30	0.90
V1V2/CaseA2 mut3	**0.62**	**(0.22 – 0.84)**	**0.05**	0.07	0.34
K169	**0.70**	**(0.17 – 0.92)**	**0.04**	0.07	0.34
PVL	**−0.67**	**(−0.88 – −0.24)**	**0.03**	0.06	0.27
**MULTIPLE PARAMETER**					
IgG (Env) & cell stain (N-U-)	**0.72**	**(0.27 – 0.91)**	**<0.001**	**<0.001**	**<0.001**
IgG (Env) & cell stain (WT)	**0.75**	**(0.29 – 0.93)**	**<0.001**	**<0.001**	**<0.001**
V1V2/CaseA2 mut3 & K169	**0.71**	**(0.27 – 0.91)**	**<0.001**	**<0.001**	**0.001**
ADCC (N-U-) & IgA/IgG (Env)	**0.45**	**(0.15 – 0.68)**	**0.02**	**0.05**	0.24
ADCC (WT) & IgA/IgG (Env)	**0.40**	**(0.13 – 0.62)**	**0.04**	0.07	0.34

*Table of linear and multivariate correlation analyses (Spearman rank) of various viral, immunologic, and clinical parameters with CD4/CD8 ratios*.

*Correlation coefficients r, 95% confidence intervals (CI), significance P and multiple-comparisons-adjusted P-values (Benjamini-Hochberg (BH) and Bonferroni method) are displayed; statistically significant results (P < 0.05) are highlighted in bold. Env, Env-specific; Neut, Neutralization breadth-potency score; N-U-, Nef- and Vpu-deficient virus infection; PVL, plasma viral load; WT, wild-type infection*.

**Table 2:** Comparison of protective host and viral factors between participants.

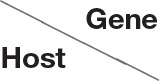	**HLA-A** **(protective)**	**HLA-B** **(protective)**	**HLA-C** **(protective)**	**CCR5Δ32**	***gag/nef* deletions and polymorphisms**
**#f1**	A^*^68:02:01G	B^*^07:02:01G	C^*^07:02:01G	Wild-type	absent
	A^*^68:02:01G	B^*^07:02:01G	C^*^07:02:01G
**#f2**	A^*^02:02	B^*^42:01:01	C^*^06:02:01G	Wild-type	absent
	A^*^30:01:01G	B^*^58:02	C^*^17:01:01G
**#m**	A^*^03:01:01G	B^*^08:01:01G	C^*^07:01:01G	Wild-type	absent
	A^*^23:01:01G	B^*^49:01:01G	C^*^07:01:01G
**#f3**	A^*^02:01:01G	B^*^15:03:01	C^*^02:10	Wild-type	**Gag T242N**
	A^*^66:01G	B^*^57:02:01	C^*^18:01

*Table summarizing between-host differences in protective host and viral factors, including HLA-I, CCR5Δ32, viral gag and nef genotypes. Genotypes are colored for each parameter according to absent (gray), weak/intermediate (pale green), and strong protective features (bold olive). For viral gag and nef, both deletions and between-host variations in adapted polymorphisms were analyzed that can be associated with delayed disease progression*.

The availability of longitudinal clinical data from time points pre-ART and pre-superinfection enabled us to calculate estimates of disease progression in the four participants (Supplementary Figure 11). Best data coverage and linear regression fits were obtained for CD4/CD8 ratios and CD4 counts with similar patterns of decrease per year (Supplementary Figure 11A). Of note, classifying disease progression based on declining clinical parameters (CD4/CD8 ratios or CD4 levels) achieved largely comparable results with clinical classifications based on ART initiation and AIDS-defining diseases in combination with absolute CD4 levels (Figure 1). Specifically, LTNP #f2 exhibited the lowest progression rate, and fast progressor #f1 exhibited the most pronounced decline of CD4/CD8 and CD4 levels. Progression rates of #m and #f3 were in between. Progressor #m who initiated ART at an earlier stage of the disease compared to slow progressor #f3 exhibited a trend to slightly lower rates of CD4/CD8 and CD4 count decline before superinfection. Correlation analyses of two study time points within 3.5 years after the estimated date of infection (EDI) and within 2.5 years post-diagnosis suggested early/intermediate predictors of disease progression. At the first study time point, high CD4/CD8 ratios and CD4 levels, as well as strong binding to cell-associated wild-type Env, predicted slower progression among the four study participants. At the second time point, strong binding to two V1V2 antigens, including V1V2/CaseA2-mut3, and lower plasma viral load indicated lower rates of disease progression.

## Discussion

Immunologic characterization of epidemiologically-linked HIV-1 transmissions has rarely been done (25–28), and most knowledge to date has been gained from studies of mother-to-child transmission (29, 30). Here we report a detailed analysis of an adult transmission cluster that, for the first time, involves viral, host, and immunologic factors that have previously been associated with protection (4, 7). Our data suggest that ADCC (N-U-) in the presence of low plasma IgA/IgG ratios and V1V2 directed antibody responses, comparable to the ones reported in the RV144 vaccine trial correlated with delayed disease progression in natural infection.

Comparing the four study participants with one another (Figures 10B,C), we observed greater composite patterns of protective viral, immune, and host parameters in individuals with slower disease progression and higher CD4/CD8 ratios. Both LTNP #f2 and slow progressor #f3 experienced beneficial clinical outcomes in association with the most potent polyfunctional responses, though their respective protective factors were differentially accentuated. Specifically, #f3 exhibited strongest ADCC (N-U-) and V1V2 binding responses in the presence of immunodominant V2 signatures, low IgA and IgA/IgG (Env) ratios, and protective HLA-I alleles. Somewhat in contrast, #f2, in the absence of strongly protective HLA-I alleles, also had substantial ADCC (N-U-) and V1V2 binding responses with low IgA and IgA/IgG (Env) ratios. However, #f2 stood out in terms of her strongest binding responses targeting both the closed (WT) and CD4-exposed (N-U-) Env, and highest Env-specific IgG levels (Figures 5, 10B,C). Multiple bidirectional transmission events between #m and #f2 may have provided strong antigenic stimulation and suggest that complex re-infections of this type may be more common than previously thought. The occurrence of superinfection at a chronic stage in #f3 suggests that the protective responses were not durable or broad enough for sustained protection, but they may have contributed to the suppression of the superinfecting virus (and the outgrowth of a recombinant). Rapid progressor #f1 had the lowest ADCC (N-U-) and V1V2 binding responses, highest Env-specific IgA/IgG ratios, and viruses with K169R mutation. This pattern is in striking contrast to LTNP #f2 from the same transmission cluster, who was infected with highly related viruses, but with K169 present in most studied viruses. It is possible that viruses in #f1 have acquired early the escape mutation K169R, associated with worse clinical outcome. Since K169 has been associated with strong immune responses (Supplementary Figure 9) (31), K169R mutations may have weakened the antibody response and associated effector functions in #f1. Among all participants, LTNP #f2 had the most beneficial clinical course of disease irrespective of the methodology used for clinical categorization (Figures 1, 2, 10, and Supplementary Figure 11) and in the absence of apparent protective host factors. Her most balanced pattern of protective antibody responses included cell-surface staining of N-U- and WT virus-infected cells and neutralization along with V1V2 responses, low Env-specific IgA/IgG ratios and ADCC against N-U- virus-infected target cells. #f2's immune response profile suggests that a delicate balance of functional immune responses, possibly against different conformations of Env is needed for the best possible control of an infecting virus. Of interest, cell-surface antibody staining did not predict/correlate with respective ADCC levels, although the same infecting virus and experimental set up was used (ADA Env WT or N-U- virus infection). These findings are in line with recent studies reporting that various parameters impact ADCC levels besides antibody binding. Other features influencing ADCC include antibody specificity, infecting strain/molecular clone, antibody orientation on the bound antigen, gp120 shedding, capacity to form multivalent antigen-antibody complexes, NKG2D ligands, and the degree of internalization of the antibody-antigen complex (32–37).

While recent macaques protection experiments suggested a redundant role of Fc-mediated effector functions for protection with the highly potent bnAb PGT121, our data support the idea that Fc-mediated effector functions do play a role in protective antibody responses that are generally inducible by natural infection or vaccination. It is also worth mentioning that IgG and IgA repertoires subtly differ between humans and non-human primates (11, 12) and protective mechanisms in macaques may not fully mirror those relevant in humans. The observed inverse correlation between ADCC (N-U-) and Env-specific IgA/IgG levels in our human study suggests that the presence of plasma IgA anti-Env Abs interfered with functional IgG-mediated responses, a finding consistent with RV144 *post-hoc* analyses (8). Of interest, ADCC (N-U-) more strongly correlated with delayed disease progression compared with ADCC (WT), indicating that the CD4-exposed or at least partially open Env conformation may play a more important role than expected in protective antibody-mediated immune responses. Accordingly, antibody-binding responses against V1V2 antigens in the presence of viral signatures in V2, which are largely occluded in the closed native trimer, correlated with better clinical outcome. These observations are in line with the results of gp120-boosted vaccination protocols such as the one used for RV144, which induced weakly-neutralizing Ab responses against open Env structures and achieved partial protection (5). Of interest, the enhanced ADCC responses in RV144 were obtained with CRF01_AE viruses (4) and subtype CRF01_AE is assumed to inherit an intrinsically more open Env configuration due to a naturally occurring H375 in the CD4 binding site (38). In contrast, the CRF02_AG related viruses of the current study carried S375, which is highly conserved across most subtypes and associated with preferably closed Env conformations (38). Recently, an asymmetric, intermediate open Env state was described, which can be induced by a CD4 mimetic and stabilized by serum Abs that are capable of mediating potent ADCC (39). At present time, we do not know whether such intermediate Env states or states comparable to the N-U- conformation exist *in vivo* and have been present as targets in the participants for a possible contribution to the observed ADCC responses. The finding that the highest ADCC (N-U-) was observed in #f3 is consistent with this participant harboring a CRF02_AG virus, which was significantly more accessible to weakly-neutralizing Abs in cell-surface staining using autologous native Env when compared to a URF virus from the transmission cluster, in which generally lower ADCC (N-U-) was observed (Supplementary Figure 4). The extent to which the studied Envs were able to engage ADCC-sensitive conformations *in vivo*, and whether these conformations were triggered in a subtype/strain-dependent manner and/or stabilized by distinct (combinations of) serum Abs, remains to be elucidated. Studies using full-length infectious molecular clones from the individuals analyzed in this study will be required to address these hypotheses.

Under the caveat of limited data points derived from four participants, this is the first report showing V1V2-directed Ab responses, elicited by viruses with specific V2 signatures (K169) being associated with clinical outcome in natural infection. Notably, our findings were obtained by studying participants in Africa and after infection with different subtypes, compared with the findings in clinical trial RV144, in which V1V2 Abs were suggested as correlates of protection (4–7). Thus, V1V2 signatures may be associated with protection against infection as well as progression, whereas favorable HLA alleles only protect against the latter (14). Results of some recent monkey experiments supported a correlation between V1V2 binding and protection (9, 10, 40), and, more generally, nnAbs have been shown to contribute to clearance of infected cells *in vivo* (41). Still, it remains unclear whether V1V2 binding and other weakly-neutralizing or nnAb-related correlates of protection are a cause or consequence of improved clinical outcome. A considerable amount of the studied immune responses may be driven by viral load or CD4^+^ cell counts resulting in mutual interference. The finding that antibody binding responses against wild-type Env (first study time point) and two V1V2 antigens (second study time point) predicted slower disease progression suggest that these responses may be markers at certain stages of the disease for other, related or unrelated, protective functional responses. The extent to what viral (Supplementary Figure 9), host [including sex and age (Supplementary Figure 10)], and cellular factors/responses (Table 2) shape the protective balance remains to be determined.

Our study highlights the extent of inter-individual variation and plasticity of immune responses after infection with highly related viral strains. While the viruses from the epidemiologically-linked cluster appeared to elicit an imprinted plasma neutralization pattern, as recently reported for transmission pairs and mother-to-child-transmission (28, 30), other immune responses differed widely among the linked individuals. Most comparable protective patterns were observed between #m and #f2, specifically for antibody binding responses, which is presumably based on the tight co-evolution of their infecting viruses as a result of numerous transmission events in both directions. Weaker responses in #m compared to #f2 in the absence of defective viral genes (*gag, env* or *nef*) or protective HLA-I or CCR5Δ32 alleles suggest a possible role of other host factors such as age and sex. Acknowledging its small size (*n* = 4), our extensive longitudinal study represents the first time that the major immune correlates of protection in RV144 were identified as being also associated with control of disease progression and maintaining CD4/CD8 ratios in natural infection. Future larger studies of natural infection and vaccine trials involving additional viral subtypes, host, and immune parameters will reveal whether the clinical correlation patterns observed in our study are generally applicable and in what extent they mirror correlates of protection in regional vaccine settings. The observed similarities between immune correlates of protection from disease progression and protection from infection underline their potential to guide vaccine research, and thus warrant further investigation.

## Materials and Methods

### Ethics Statement

This study was carried out in accordance with the recommendations from the Institutional Ethical Review Board of the New York University School of Medicine and the National Ethics Committee of Cameroon's Ministry of Public Health. All study participants were Cameroonian adults. All subjects gave written informed consent in accordance with the Declaration of Helsinki, before inclusion in the study. The protocol was approved by the Institutional Ethical Review Board of the New York University School of Medicine and the National Ethics Committee of Cameroon's Ministry of Public Health.

### Study Design and Participants

The observational study focused on a heterosexual HIV-1 transmission cluster with the goal to identify immune parameters associated with clinical outcome after infection with related strains. Four HIV^+^ individuals, three females (#f1, #f2, and #f3) and one male (#m) from Yaoundé, Cameroon, were longitudinally studied from 2002 to 2017. Participants #m, #f1, and #f2 are epidemiologically-linked in a polygamous heterosexual relationship (both reported and genetically confirmed). Participant #f3 is a non-linked HIV^+^ case from the same cohort who was followed up over the same 15-years study period. She was selected based on similar dates of infection (2001–2003), baseline CD4 counts (346-1206 cells/μL), and infection with a related HIV-1 subtype (CRF02_AG vs. URF02_AG/F2) compared to the linked participants. The decision to include female #f3 in the study was made before the initiation of the immune correlate analyses. In the study questionnaire, #m indicated polygamous heterosexual orientation within a marriage with two females, whereas #f1, #f2, and #f3 listed monogamous heterosexual relationships. No clinical data or questionnaire entries suggested additional transmissions from outside partners, which supported the phylogenetic findings of the study.

After a positive HIV screening result in 2002/2003, the four HIV^+^ volunteers donated blood at the Medical Diagnostic Center (MDC) in Yaoundé, Cameroon, from 2002 to 2017. Whole blood was shipped from Yaoundé to NYUSoM, New York, NY, where plasma and peripheral blood mononuclear cells (PBMCs) were separated using Ficoll gradient centrifugation (Histopaque, Sigma-Aldrich, St. Louis, MO, USA), and subsequently stored at −80°C. Participants #m and #f1 experienced standard and rapid progressive courses of HIV disease, respectively, based on their CD4 counts dropping below 300 cells/μL (#m) or repeatedly below 200 cells/μL (#f1) within 4 years after infection. In contrast, participant #f2 was classified as a long-term non-progressor (LTNP), based on CD4 counts >500 cells/μL in the absence of AIDS symptoms for >8 years without ART. Participant #f3 exhibited a slow progression without AIDS symptoms or ART for >8 years (42).

Sampling started while all participants were ART-naïve. ART was initiated based on regional treatment guidelines per period: before 2004: no ART publicly available; 2004–2009: ART initiation when CD4<200 cells/μL; 2010–2013: CD4<350 cell/μL, 2014–2015: CD4<500 cell/μL; starting 2016: ART available to all HIV^+^ patients regardless of CD4 counts. The four study participants were initially included in the screening for dual infections using a heteroduplex assay (43), and were renamed for simplicity reasons: #f1 and #f2 referred to #6541 and #6542, respectively, and both were described as singly infected. #f3 referred to #6506 and was introduced as superinfected. #m referred to #6544, who was initially assumed to be superinfected (this, however, could not be confirmed in the current study).

Immune response, phylogenetic, and host factor analyses were performed by five geographically distant working groups to provide independent and, in major parts, blind data generation, processing, and primary data analyses.

### CD4 and CD8 Counts

CD4 and CD8 cell counts were measured by FACSCount (Becton Dickinson), Guava Easy Cyte systems (CD4 counts, starting 2011) (Millipore-Sigma), or FACSCanto and Tritest Reagent (#f3's CD4/CD8 ratios, 2012–2017) (Becton Dickinson). CD4/CD8 ratios were used as an established marker for clinical outcome and HIV-related immune dysfunction (44). Longitudinal CD4/CD8 bump charts (sorted stream graphs) were created using RAWGraphs (Sankey interpolation; padding set to zero) (http://app.rawgraphs.io/). Bump charts were sorted in descending order of the participants' CD4/CD8 ratios.

### Viral Load

Viral load was determined using the Abbott m2000 RealTime HIV-1 assay as per the manufacturer's instructions (Abbott Molecular), which has been demonstrated to assess CRF02_AG samples accurately. Briefly, the m2000 RealTime HIV-1 assay performs automated extraction (input volume of 0.6 mL, m2000sp apparatus), real-time polymerase chain reaction (PCR) amplification of the integrase gene fragment, and noncompetitive fluorescent detection (m2000rt instrument, dynamic range of 40–107 copies/mL).

### Incidence Testing

To distinguish chronic (≥6 months) and acute (<6 months) infections, a multi-assay algorithm was used including the HIV-1 LAg-Avidity assay (HIV-1 LAg-Avidity EIA, SEDIA Biosciences Corporation) and the BioRad-Avidity Assay based on the Genetic Systems 1/2+O ELISA (Bio-Rad Laboratories) (45, 46). Both assays were performed according to the manufacturer's instructions. Briefly, for the Lag-Avidity assay, plasma was incubated for 60 min at 37°C with the HIV-1 antigen (rlDR-M). Disassociation buffer was added to remove Abs with low avidity, and goat anti-human IgG-HRP was added to detect bound IgG. TMB substrate was used to initiate a color-change reaction, and its intensity was measured as optical density. The BioRad-Avidity Assay was carried out in the same fashion but with the following modifications: the initial incubation was carried out at 4°C, and diethylamine was used as a chaotropic agent to disrupt binding of Abs with low avidity. Results from both assays were compared with the established internal controls to calculate the duration of infection.

### RNA Extraction and cDNA Synthesis

Strict precautions were taken to exclude unspecific amplification and cross-contamination, which included the separate processing of samples from different participants, performing related PCR assays at different times, negative controls, frequent cleaning, and nucleotide removal treatments, as well as performing phylogenetic control analyses across the cohort and recently studied samples.

Before RNA extraction, the virus in 500 μL of plasma was concentrated by centrifugation at 14,000 x g for 1 h at 4°C. After removal of 360 μL supernatant, the virus pellet was re-suspended in the remaining 140 μL of supernatant by vortexing and viral RNA was extracted using the QIAamp Viral RNA Mini kit according to manufacturer's instructions (Qiagen) (46). The generation of cDNA was done using SuperScript III (Thermo Fisher Scientific) or GoScript (Promega) Reverse Transcriptase according to the manufacturer's instructions.

### Genomic and Proviral DNA Extraction

DNA was extracted from frozen PBMCs using the QIAamp DNA Mini Kit (Qiagen) according to manufacturer's instructions.

### Single Genome Amplification (SGA), *env* PCRs

Single genome amplifications (SGA) were performed according to established protocols covering the full *env* gene (HIV region 5954–9174, HXB2 numbering) (47). Nested PCRs were performed on endpoint-diluted cDNA using high-fidelity PrimeSTAR GXL DNA polymerase (Clontech). Amplicons resulting from template cDNA dilutions yielding <30% positive PCR reactions were assumed to be based on single genomes. Primers were used as published (47); however, instead of EnvN, the primer 02AG-EnvN was used, optimized for Cameroonian sequences (02AG-EnvN: 5′-GTTCTGCCAATCTGGGAAGAATCCTTGTGTG-3′) (48).

Additionally, nested PCRs were performed over a shortened version of *env*, HXB2 region 6225-7838 (including full gp120) (46), which enabled the characterization of viral sequences that could not be amplified using the “full-*env*” SGA primers: first-round PCR using PrimeSTAR GXL DNA polymerase (Clontech) with primers EnvA (5′-GGCTTAGGCATCTCCTATGGCAGGAAGAA-3′) and gp120out (5′- GCARCCCCAAAKYCCTAGG-3′), second-round PCR using Platinum Taq polymerase (Life Technologies) with primers EnvB (5′-AGAAAGAGCAGAAGACAGTGGCA-3′) and gp120in (5′-CGTCAGCGTYATTGACGCYGC-3′).

### Env Cloning, Colony PCR, and Sequencing

PCR products were cloned into pcDNA3.1 (SGA full-*env*) (47) or pCR4 TOPO (shortened-*env*) (Life Technologies) and transformed into One Shot TOP10 competent *E.coli* or MAX Efficiency Stbl2 competent cells (Life Technologies). Screening for positively transformed *E.coli* colonies was performed by colony PCR using Phusion 2x Master Mix (Thermo Scientific), universal vector-specific primers M13F/M13R (pCR4 TOPO) or T7/BGHrev (pcDNA3.1), and colonies diluted in 100 μL LB medium (0.5 μL used as template for PCR). LB_Amp_ cultures of positive clones were grown overnight; plasmids were isolated using the QIAprep Spin Miniprep kit (Qiagen). Plasmids were sequenced for the insert using vector-specific primers. Sequence analysis and assembly were performed using SeqMan Pro (DNASTAR). For subsequent phylogenetic and epitope analysis, all individual sequences per longitudinal *env* time point and study participant were averaged to consensus (con) sequences using Consensus Maker (Los Alamos National Library (LANL) Database) (www.hiv.lanl.gov) or SeqMan Pro.

### Phylogenetic and Recombination Analysis

Sequence alignments were performed using MUSCLE (MEGA5.2) with HIV-1 reference sequences including circulating recombinant forms (CRFs) from the LANL HIV sequence database. Neighbor-joining phylogenetic trees were created using MEGA5.2 (Kimura 2-parameter model, 200–500 bootstrap replications) and FigTree1.4.3 (49, 50). Genetic distances between sequences were calculated in MEGA5.2 (Kimura 2-parameter model) (46). Subtyping was based on phylogenetic (HIV BLAST, www.hiv.lanl.gov) and recombination analyses (Simplot3.5.1) (9, 51) of the *env* sequences. Also, BootScan and SimPlot tools were used to identify possible recombination events and their breakpoint regions (9, 51). The window width, step size, and bootstrap replicates were set to 200 bp, 20 bp, and 100, respectively. The proportional amino acid contribution per site within a set of aligned protein sequences was determined using WebLogo (https://weblogo.berkeley.edu/). Potential N-linked glycosylation sites were determined using the N-Glycosite tool from the Los Alamos HIV sequence database (http://www.hiv.lanl.gov/) on Env sequence alignments.

### BEAST (Bayesian Evolutionary Analysis by Sampling Trees)

BEAST was used to reconstruct the likely direction and timing of transmission events and to estimate dates of infection. The data set consisted of 564 longitudinal, functional viral envelope sequences (HXB2 region 6225-7838) sampled between 2002 and 2017 from four HIV^+^ individuals. Two separate alignments were performed for the linked HIV^+^ individuals (#f1, #f2, #m; *n* = 402) and for the incidence-matched case (#f3; *n* = 162). The best-fitting nucleotide substitution model was identified using MEGA 5.02 and applied in the reconstruction of time-calibrated phylogenetic histories in a Bayesian statistical framework implemented in the software package BEAST v1.8.4. BEAST uses Markov chain Monte Carlo (MCMC) sampling including information on the dates and “locations” (= participant) when and “where” the viruses were sampled to infer the most probable pathways of past transmission events that gave rise to the observed contemporary distribution among individuals (52–54). BEAST analyses were performed using the BEAGLE likelihood calculation library, to increase computation speed (55, 56).

#### Sequence Evolution

To accommodate among-lineage rate variation, we applied an uncorrelated relaxed molecular clock that models branch rate variation according to a lognormal distribution for its high accuracy and precision (57). For inference of the historic population dynamics, a flexible non-parametric Bayesian skygrid tree prior was applied, which has been shown to outperform other non-parametric coalescent priors for divergence time estimation based on simulation data (58).

The best fitting nucleotide substitution model for the alignments identified by MEGA was the general time reversible model (59) with gamma-distributed rate heterogeneity with four categories (GTR + G_4_) (60). The parameter estimates of the posterior median nucleotide substitution rate were 9.27 × 10^−3^ substitutions/site/year (95% HPD = 7.98 × 10^−3^-1.05 × 10^−2^, linked HIV^+^ individuals), and 1.01 × 10^−2^ substitutions/site/year (95% HPD = 8.61 × 10^−3^-1.18 × 10^−2^, incidence-matched case). These rates are similar to the intra-host HIV-1 rates estimates reported previously of 1.58 × 10^−2^ (9.99 × 10^−3^-2.04 × 10^−2^) substitutions/site/year within *env* gp120 V1C5, estimated from a longitudinal cohort of 32 individuals infected with a single viral variant (61).

The estimated mean time to the most recent common ancestor (TMRCA) of the viruses from the epidemiologically-linked individuals was 1999 (95% HPD = 1995–2002) and the most likely root state among those considered was participant #m. The estimated mean TMRCA of the viruses from #f3 was 2002.3 (95% HPD = 2002.0-2002.4).

#### Discrete Phylogeography Diffusion Models

To model the historic diffusion dynamics of the URF viruses among the three linked HIV^+^ individuals we used a reversible continuous-time Markov chain (CTMC) process with a Bayesian stochastic search variable selection (BSSVS) to quantify the statistical support for different movement pathways in the form of Bayes factors (BF). A BF>3 is indicative of substantial support, and BFs>10 and >100 are indicative of strong and decisive support (56, 62). To quantify the magnitude of these transmission events (“Markov jumps”), and the time spent in each individual host (“Markov rewards”), we used stochastic mapping techniques (63, 64) and the BEAGLE library.

For both data sets, ten independent MCMC runs of 600 million states in length were performed in BEAST. The maximum clade credibility (MCC) trees were annotated with TreeAnnotator (BEAST package), and the BF support values were calculated using SpreaD3 v0.9.6 (65).

### *Gag* and *nef* Sequence Analysis

Viral *gag* and *nef* sequences were studied for between-host variations of deletions and adapted polymorphisms that are known to be able to influence HIV-1 disease progression (66–68). For each participant, *gag* and *nef* bulk sequencing was performed using a study time point pre-ART and pre-superinfection (#f1: 6, #f2: 5, #m: 4, and #f3: 9). PCRs were performed with PrimeSTAR GXL DNA polymerase (Clontech) as described above. *Gag* PCRs (HXB2 region 890-2278) were run with in-house first-round primers Gag2_For (5′-GACTAGCGGAGGCTAGAAG-3′) and Gag1_Rev (5′-CCAATTCCCCCTATCAT-3′), as well as second-round primers Gag4_For (5′-TAGTATGGGCAAGCAGGGA-3′) and Gag3_Rev (5′- GGTCGTTGCCAAAGAGTGA−3′). *Nef* PCRs (HXB2 region 7818-9567) were run with in-house first-round primers Gp120in_For1 (5′-CAGCAGGAAGCACTATGGGCG-3′) and 3′UTR_Rev1 (5′- TATTGAGGCTTAAGCAGTGGGTTC-3′), as well as second-round primers Gp120in_For2 (5′-GCRGCGTCAATRACGCTGACG-3′) and 3′UTR_Rev4 (5′-GCTCAAATCTGGTCTAGCAAGAGAGA-3′).

### IgG Antibody Isolation From Plasma

Plasma samples were heat inactivated for 1.5 h at 56°C. IgG was isolated from 500 μL heat-inactivated plasma using 450 μL Protein G Sepharose 4 Fast Flow (GE Healthcare Life Sciences) as previously described (46, 69).

### Production and Titration of HIV-1 Pseudoviruses

*Env* plasmids SV-A-MLV-*env*, HIV-1 strain Q23 ENV17 (clade A), 250 (clade CRF02_AG), and ZM249M.PL1 (clade C) were obtained through the NIH AIDS Reagent Program (NIH); X2131_c1 (clade G) from Dr. Michael Seaman (BIDMC Harvard); pCAGGS_JR-FL.JB (clade B) from Dr. John Mascola (NIH); and P2059-B12 (clade F2) from Dr. David Montefiori (Duke University). The desired *Env* plasmid was combined with pSG3ΔEnv backbone at optimized ratios (mostly 1:3) (46, 70) and transfected into 293T/17 cells (ATCC® CRL-11268™) using polyethylenimine (linear PEI 25 K, Polysciences). After 48 h incubation, the supernatants were collected, filtered through a 0.45-micron filter, and stored at −80°C until use. Titration was carried out in TZM-bl cells for 48–72 h using serial dilutions of 25 μL virus, according to the Montefiori protocol (29, 70). Luminescence was detected by adding Bright Glo Reagent (Bright-Glo Luciferase Assay System, Promega) to each well for ~2 min and measuring the relative light units (RLUs) on a Victor3 Multilabel Counter (PerkinElmer). In order to provide comparable results between the high-titer reference and lower titer, primary autologous pseudoviruses, virus titers were adjusted to ~20,000 RLUs per well. Subtype F2 (P2059_B12) pseudoviruses exhibited the lowest titers yet were used in titers of at least 20x background signals.

### TZM-bl Neutralization Assay

Neutralization was reported as a reduction of single-round infection-induced luciferase expression after 48–72 h incubation (29, 70). Neutralization assays were carried out in duplicate and experiments were repeated at least twice with plasma or done once or twice with IgG, according to IgG availability. TZM-bl cells were maintained in Dulbecco's modified Eagle's medium (DMEM with L-glutamine, sodium pyruvate, glucose, and pyridoxine; Gibco-Thermo Fisher Scientific), supplemented with 10% fetal bovine serum (FBS) (Thermo Fisher Scientific), 2.5% HEPES (Gibco-Thermo Fisher Scientific), and 1% Penicillin/Streptomycin (Lonza BioWhittaker) and passaged twice a week. Serial dilutions of heat-inactivated plasma (1:20–1:320) or IgG (500–0.1 μg/mL) were mixed with ~20,000 RLUs of titrated pseudovirus in half-area flat-bottom 96-well plates (Costar). After 30 min incubation at 37°C, 5,000 TZM-bl cells in 50 μL DMEM media containing 10 μg/mL DEAE-dextran were added to each well. The optimal concentration of DEAE for reaching maximum infection was determined for each lot by titration of a 5 mg/mL stock solution. The plates were incubated 48–72 h at 37°C and measured as described above. After subtraction of background luminescence, percent neutralization was calculated by division of the mean RLU for each set of duplicates by the mean RLU in the respective replicates of virus-only control wells, multiplied by 100 (29, 70). Mean neutralization (%) and standard deviation were determined for each data point. Negative mean neutralization values were set to zero for clarity. Neutralization curves are shown as nonlinear regression fits (least squares ordinary fit, one-phase decay, GraphPad Prism). IC_50_ values were determined in the fitted curves for the reciprocal plasma dilutions or IgG concentrations at 50% neutralization and illustrated in heat maps.

#### Breadth-Potency

Relative breadth-potency scores were determined within each study sample using an established normalization algorithm under consideration of the range and average of IC_50_ values of the study samples and pseudoviruses used. Briefly, for each pseudovirus that achieved at least one detectable IC_50_ score (IC_50_ >20) for the longitudinal plasma samples studied (i.e., all except C.ZM249), a mean neutralization sensitivity score was calculated by averaging all determined IC_50_ values for the four participants (IC_50_ <20 was set to zero). If the IC_50_ for a given plasma-virus pair was higher than the mean IC_50_ of the four individuals, then a score of 1 was given, while those below the mean were scored as 0. A composite breadth score was calculated for each plasma sample (sum). Potency scores were calculated by dividing the IC_50_ value for a given plasma-virus combination by the mean IC_50_ for the particular virus. An overall potency score was calculated for each plasma sample by averaging the potency scores derived from the applicable viruses. Combined breadth-potency scores were calculated by the multiplication of the overall breadth and potency scores per plasma sample (71, 72).

### Antibody-Dependent Cellular Cytotoxicity (ADCC)

ADCC experiments were performed in CEM.NKR cells infected with replication-competent virus (NL4-3 backbone with ADA Env and IRES/GFP cassettes; NL4.3.ADA.IRES.GFP) (18). Experiments were done using VSV-G pseudotyped wild-type (WT) as well as Nef- and Vpu-deficient (N-U-) virus, the latter is known to enhance the susceptibility of infected cells to ADCC by impeding Nef- and Vpu-mediated CD4 downregulation on the surface of HIV-infected cells (18, 21, 23, 73). CEM.NKR target cells were mixed with PBMC effector cells (isolated from healthy HIV-negative donors) at an effector/target (E/T) ratio of 10:1. Heat-inactivated patient sera were added at 1:1,000 dilution; A32 mAb (5 μg/mL) served as positive control. ADCC was determined in killing assays based on the flow cytometric reduction of GFP signals in cells incubated with heat-inactivated plasma/mAb vs. mock (18).

### Cell Surface Staining

Plasma antibody or mAb binding to trimeric patient Env was analyzed by Env cell-surface expression on CEM.NKR infected cells (ADCC experiments), or 293T cells (ATCC® CRL-3216™) transfected with SGA full-*env* pcDNA3.1 plasmid and 0.5 μg pIRES-GFP with/without 0.5 μg of a pcDN3.1 vector expressing human WT CD4, and flow cytometric analysis (18, 74). Env-expressing cells were detected using plasma diluted 1:1,000 or anti-Env mAbs (5 μg/mL) PGT151 (IAVI), PG9 (Polymun), VRC03, CH58, CH59 (75), 17b, A32, 19b, 7B2, F240, and 830A (NIH AIDS Reagent Program), and secondary antibody goat anti-human coupled to Alexa Fluor 647 (Invitrogen).

### IgA & IgG Quantitation

Total IgA and IgG quantitation were performed with the Bethyl Laboratories Inc Human IgA and IgG ELISA Quantitation Sets. The ELISA Starter Accessory Kit contained the Microtiter plates as well as all necessary buffers for the quantitation. For HIV-specific IgA and IgG determination, microtiter plates were coated with HIV-1 recombinant envelope glycoprotein (gp120 JRFL) at 1 μg/mL instead of the capture antibody provided by the kit. Dilutions of the plasma tested were as follows: total IgG (1:100,000), HIV-specific IgG (1:10,000), total IgA (1:5,000), HIV-specific IgA (1:10). The diluent for dilutions consisted of TBST + 0.1% Triton-X. IgG and IgA concentrations were determined via interpolation from the standard curve (GraphPad Prism 7, Sigmoidal, 4PL, X is log (concentration), nonlinear fit).

### Multiplex Bead-Based xMAP Assay (Luminex)

#### Preparation of Antigen-Coated Microspheres

Multiplex binding analyses were performed with a set of antigens that have been recently characterized. They were selected based on cross-reactivity with sera from HIV-1 infected/vaccinated individuals from Thailand, USA and Cameroon (46, 76, 77). HIV antigens were conjugated to magnetic beads as described with minor changes. Antigens (*n* = 11) included recombinant gp120 from different clades, peptides (V3, C5, V2), and constrained and unconstrained V1V2-scaffold proteins bearing V1V2 Env inserts from a variety of HIV-1 strains and clades (76, 78, 79). V1V2/ZM109-TTB, V1V2/ZM109-1FD6, and V1V2/ZM53-2F5K were provided by X. Kong (NYU), V1V2/1086-tags by H. Liao (Duke University), V1V2/Case A2 gp70 and V1V2/Case A2 mut3 by A. Pinter (Rutgers University). V1V2/Case A2 mut3 (clade B) included three mutations in the V2 immunodominant region, i.e., V169K, E172V, and E173H, designed to better match the antigen with the A244 strain (clade CRF01_AE) from the RV144 vaccine trial (24). Scaffolds for constrained V1V2 antigens were initially screened for efficient engraftment of diverse V1V2 proteins (76). From the available list, one monomeric, one trimeric, and one pentameric V1V2 scaffold protein with reported strong antigenic properties were chosen for the current study. All peptides were used with an N-terminal 6x Lys-Gly-linker, to ensure sufficient coupling of small molecules to the beads. The clade C consensus V3 linear peptide (KGKGKGKGKG-NNTRKSIRIGPGQTFYATGDIIG) and the clade E cyclic V2_92TH023_ (cV2) peptide (KGKGKGKGKGKG-CSFNMTTELRDKKQKVHALFYKLDIVPIEDNTSSSEYRLINC) were purchased from GenScript, and the C5_ZM109_ linear peptide (KGKGKGKGKGKG-VEIKPLGIAPTEAKRRVVQREKR) was purchased from BioPeptide. Gp120_ZM53_ (#IT-001-RC8p) and gp120_MG505_ (#IT-001-101p) were obtained from Immune Tech.

Covalent coupling was performed in a two-step carbodiimide reaction, using the xMAP Antibody Coupling (AbC) Kit (Luminex) according to the manufacturer's instructions. Briefly, carboxylated xMAP beads (Luminex) were coupled to 0.5 μg protein/million beads (V1V2/ZM109-TTB and V1V2/ZM109-1FD6) or 1 μg protein/million beads (all peptides) or 4 μg protein/million beads (all gp120s, V1V2/ZM53-2F5K, V1V2/1086-tags, V1V2/Case A2 gp70, V1V2/Case A2 mut3, and BSA). These concentrations were determined by titration of the antigens against a control mAb pool and a control serum sample [(see b) xMAP bead assay]. For the coupling reaction, the bead storage buffer was removed from the beads by magnetic separation, and beads were washed with Activation Buffer (Luminex). For reactions containing up to 5 × 10^6^ beads per sample, 10 μL each of 50 mg/mL N-hydroxysulfosuccinimide (Sulfo-NHS) and 1-ethyl-3-(3-dimethlyaminopropyl)carbodiimide-HCl (EDC) were added and incubated for 20 min at room temperature. Activated beads were washed twice with 250 μL Activation Buffer and resuspended in 100 μL Activation Buffer. Antigen was then added and incubated for 2 h with rotation. Coupled beads were washed three times with 1 mL Washing Buffer (Luminex) and resuspended in 500 mL PBS-TBN (PBS, 0.1% BSA, 0.02% Tween-20, 0.05% Azide, pH 7.4). Finally, the coupled beads were counted, diluted to a concentration of 500,000 beads/mL and stored at 4°C for up to 1 month prior to use.

#### xMAP Bead Assay

First, a bead mixture was prepared by adding each bead type at 50 beads/μL in PBS-TBN. 50 μL/well (2,500 beads of each bead type/well) were aliquoted from this mixture into black, clear bottom 96-well plates (Greiner Bio-One). Fifty Microliter serum samples were added at a dilution of 1:200 and incubated with the bead mixture for 1.5 h at room temperature in the dark with shaking. Wells were washed twice with 100 μL/well PBS-TBN and incubated with 100 μL/well of either biotinylated anti-human IgG (4 μg/mL) (Abcam), biotinylated anti-human IgG1 (4 μg/mL), IgG2 (1 μg/mL), IgG3 (3 μg/mL), or IgG4 (4 μg/mL) (SouthernBiotech) for 30 min at room temperature in the dark with shaking. After washing twice with 100 μL/well PBS-TBN, samples were incubated with 100 μL 1 μg/mL Streptavidin-PE (BioLegend) for 30 min at room temperature in the dark with shaking. Wells were then washed twice with 100 μL/well PBS-TBN and beads measured for PE fluorescence using a Luminex FlexMAP3D device with xPONENT 4.2 software. Plasma samples were tested in duplicate, and results are shown as mean fluorescent intensity (MFI). Beads coupled to BSA and serum from an uninfected donor served as negative controls. A cocktail of IgG1 and IgG3 mAbs composed of multiple V2 (697, 830A, 1393A, and CH58), V3 (3869), and C5 (670, 1331A) mAbs (Zolla-Pazner and Gorny laboratories) were used in each experiment for inter-experimental standardization. Also, a mAb pool of IgG1 (697, 1393A, CH58, 3869, and 670) and/or IgG3 only (830A and 1331A) was run for each experiment to determine cross-IgG subclass binding.

#### Data Analysis

Background correction was performed by subtracting the highest MFI background signal (PBS-TBN, IgG1+IgG3, IgG1, or IgG3 mAb pool binding signals, dependent on the studied IgG subclass) from the sample MFI signal. Heatmaps were generated in GraphPad Prism 7.03.

Nonlinear regression fits were calculated and concentrations at half-maximal binding (EC50) determined (GraphPad Prism). A standard ELISA protocol was followed; the antigens (1 μg/mL) were adsorbed onto ELISA plates (Immulon 4HBX; Thermo Fisher, Waltham, MA) overnight at 4°C. Plates were blocked with 3% BSA diluted in PBS/0.05% Tween-20 for 1 h at room temperature (200 μL per well), to reduce non-specific binding. Plasma samples were analyzed at 1:100 dilutions, IgG samples at 1:5 serial dilutions starting with 500 μg/mL diluted in 0.1% Triton-X, for 1.5 h at 37°C. Alkaline phosphatase-conjugated anti-human IgG (1:2,000; Southern Biotech, Birmingham, AL) was used as a secondary Ab. P-nitrophenyl phosphate tablets (Thermo Fisher, Waltham, MA) dissolved in diethanolamine (Thermo Fisher, Waltham, MA) were used as the substrate. The optical density was read on a microplate reader (Tecan Sunrise) at 405 nm.

### ELISA

Plasma-purified IgG binding experiments were performed against a set of HIV-1 gp120 and gp41 antigens, including a scaffolded V1V2 protein (V1V2/ZM109-1FD6), (46, 78) a cyclic V3 peptide (V3/ZM109), (49, 80) gp120core JRFL, (50, 81) BG505 SOSIP, (51, 82) and an MPER gp41/con B peptide (NIH AIDS Reagent Program) as previously described (46). Additional V1V2-fusion proteins used in ELISAs with plasma (1:100) were either obtained from X. Kong (NYU) (76) or purchased from Immune Technology. Controls gp120 MN and gp41 MN were obtained through Immune Technology or the NIH AIDS Reagent Program (NIH), respectively. Plasma or plasma-purified IgG from a Cameroonian HIV-1 uninfected individual was included as a negative control. Standard ELISA protocols were followed (46, 83).

### Host Factor Analysis

#### HLA Class I Typing

Sequence-based typing (SBT) for HLA-A, B, and C was performed on DNA extracted from frozen plasma (for #f3, additionally from PBMCs) (Supplementary Table 2). Two or three longitudinal time points were studied per participant for confirmation and to exclude sample mix-up. Independent nested polymerase chain reactions (PCR) were used to amplify ~1,000 bp regions spanning Exon 2 and 3. PCR reactions using universal locus-specific primers and subsequent sequencing were done according to established protocols (84). HLA protective assessment was done according to recent studies (85–87).

#### Screening for CCR5Δ32 Mutations

Analysis for CCR5Δ32 mutants was done using two longitudinal time points per participant based on established protocols. The CCR5 region of interest was amplified by PCR using specific CCR5Δ forward (for) (5′-ACC AGA TCT CAA AAA GAA GGT CT-3′) and CCR5Δ reverse (rev) (5′-CAT GAT GGT GAA GAT AAG CCT CAC A-f3′) primers. Gel electrophoretic analysis with the use of a 2% agarose gel enabled to distinguish the presence of homozygous WT alleles (single band at 225 bp), homozygous CCR5Δ32 mutant alleles (single band at 193 bp), and heterozygous CCR5WT/Δ32 alleles (225 and 193 bp bands).

### Software Scripts for Graphic Illustrations

Correlograms were generated using the corrplot package and chord diagrams based on the circlize and ComplexHeatmap package in R and RStudio (v1.1.423) (88). Radar plots were created in Microsoft Excel 2010 with manual normalization and, if applicable, inversion of data.

### Statistics

D'Agostino & Pearson (*n* ≥ 8) or Shapiro-Wilk (3 ≤ *n* ≤ 8) normality tests (Prism) were performed to analyze whether the values come from a Gaussian distribution and thus to determine the appropriate statistical test to be used (parametric or non-parametric). Statistical comparisons of individual or grouped datasets with normal distribution (parametric) were made using unpaired, two-tailed *t*-tests or one-way ANOVA tests (GraphPad Prism v7.03) with Tukey multiplicity adjustment. Non-parametric comparisons were made using Kruskal-Willis tests with Dunn's correction for multiple comparisons. Correlation analysis was done using non-parametric Spearman rank tests. Correlation coefficients *r*, 95% confidence intervals, and *P*-values were calculated in R software (v3.4.3) or Prism. Linear regression fits were done in Prism. The site-scanning analysis was done for all non-conserved amino acid sites for the given data set and genomic region using functional Env sequences. Two-tailed Spearman rank tests were done for the selected sites correlating the presence of the major residue per site with immunologic or clinical parameters. Multivariate analysis was performed using Spearman-like canonical correlations of ranks. Corresponding *P*-values were calculated using the Fisher transformation (R). Corrections for multiple comparisons were done in R using Benjamini-Hochberg (BH) and Bonferroni methods. The two adjustment methods are based on different algorithms and vary in the degree of stringency. BH takes into consideration the ranking of data points and is accurate to adjust for multiple comparisons based on non-parametric Spearman rank correlations as applied in our study. Bonferroni is a simple, frequently used correction method providing more stringent adjustments irrespective of ranks.

## Data Availability

SGA *env* and consensus *env* sequences are available from GenBank with the accession numbers MH460464- MH460513. Whole sets of *env* sequences are available upon request. Correspondence, data and material requests should be addressed to Ralf Duerr (Ralf.Duerr@nyumc.org).

## Ethics Statement

This study was carried out in accordance with the recommendations from the Institutional Ethical Review Board of the New York University School of Medicine and the National Ethics Committee of Cameroon's Ministry of Public Health. All study participants were Cameroonian adults. All subjects gave written informed consent in accordance with the Declaration of Helsinki, before inclusion in the study. The protocol was approved by the Institutional Ethical Review Board of the New York University School of Medicine and the National Ethics Committee of Cameroon's Ministry of Public Health.

## Author Contributions

PN and RD conceived and designed the study. MiT, JB, AB, SD, GH, SW, VI, AD, SP, SS, LM, and XW performed the experiments. MaT, GH, SW, AN, ZB, AR, AF, PN, and RD conducted experimental and statistical data analysis. JM, JT, X-PK, AN, DK, AJN, TQ, SZ-P, and MG provided technical and supervisory support. RD drafted the original manuscript. GH, SW, DK, ZB, TQ, SZ-P, AR, AF, and MG participated in the editing and review of the manuscript. All authors reviewed the manuscript for intellectual content and approved the final version for publication.

### Conflict of Interest Statement

The authors declare that the research was conducted in the absence of any commercial or financial relationships that could be construed as a potential conflict of interest.
