# Supplementary material for: Immune Correlates of Disease Progression in Linked HIV-1 Infection
Source: Front Immunol. 2019 May 14;10:1062. doi: 10.3389/fimmu.2019.01062 (PMC6527802; doi:10.3389/fimmu.2019.01062)
Supplement: Supplementary file 1 [file Presentation_1.pdf]

Supplementary Material

Supplementary Figures

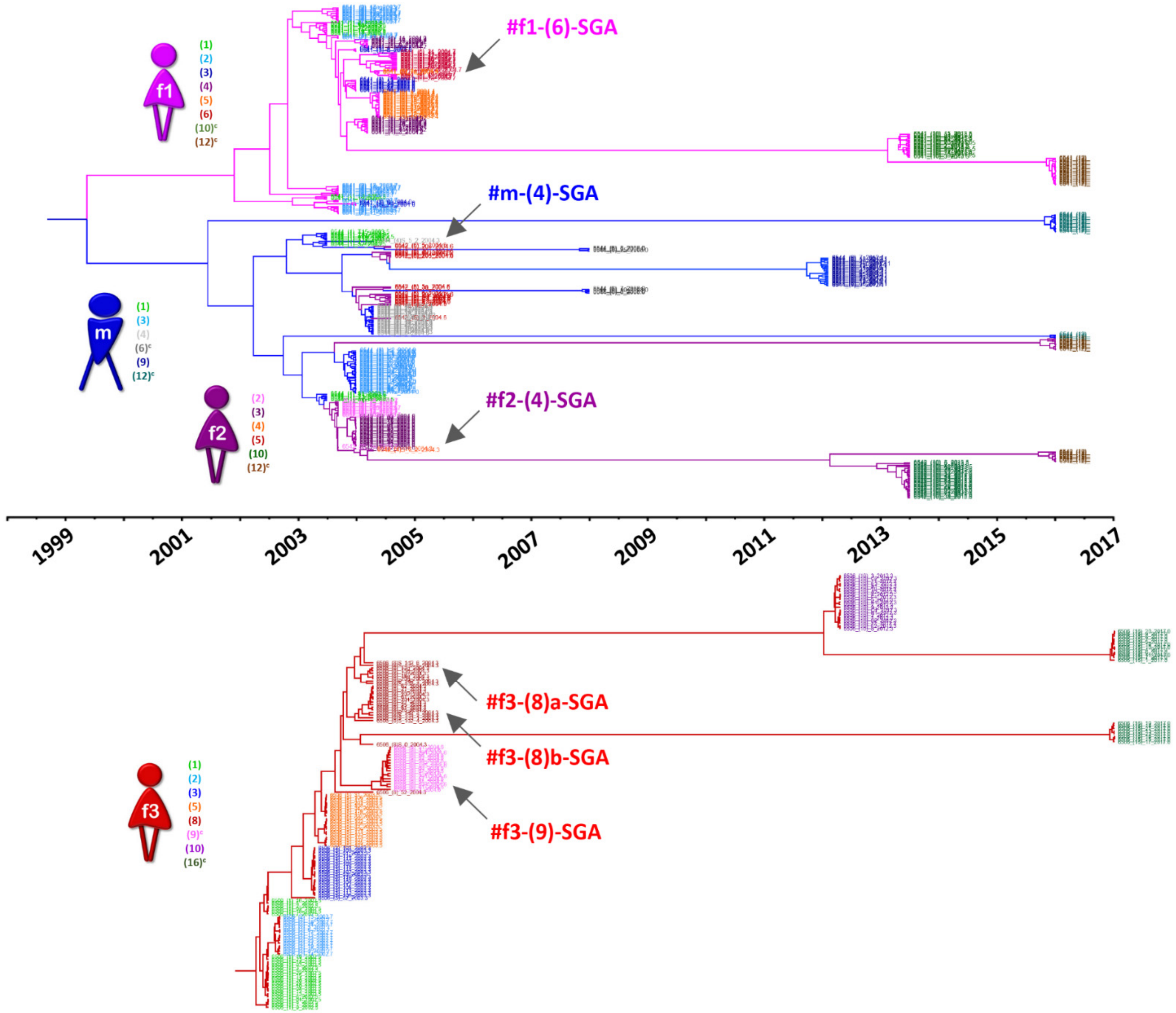

Supplementary Figure 1 | Longitudinal phylogenetic analysis of participants' *env* sequences using time-calibrated trees in BEAST. Viral (co-)evolution of four study participants was determined in BEAST time-calibrated MCC trees using 564 functional *env* sequences from the years 2002–2017 (HIV region 6225–7817 according to HXB2 numbering). Two trees are shown separated by a common timeline. The upper tree displays the viral evolution in a transmission cluster from Cameroon composed of three individuals infected with HIV-1 URF viruses; the lower tree displays the viral evolution in an incidence-matched control case from the same study cohort infected with a CRF02_AG virus. The branches of the trees are color-coded according to each individual. The taxa are color-coded according to time point as indicated in the legend to the right of each participant symbol.

Six SGA *env* sequences that were selected for detailed genetic and functional analyses are indicated with gray arrows and labeled, *i.e.*, three representatives of the bulk of URF sequences from #f1, #f2, and #m, as well as three representatives of the bulk of CRF02_AG sequences from #f3 before initiation of antiretroviral treatment and occurrence of superinfection. BEAST: Bayesian evolutionary analysis by sampling trees; MCC: maximum clade credibility; URF: unique recombinant form.

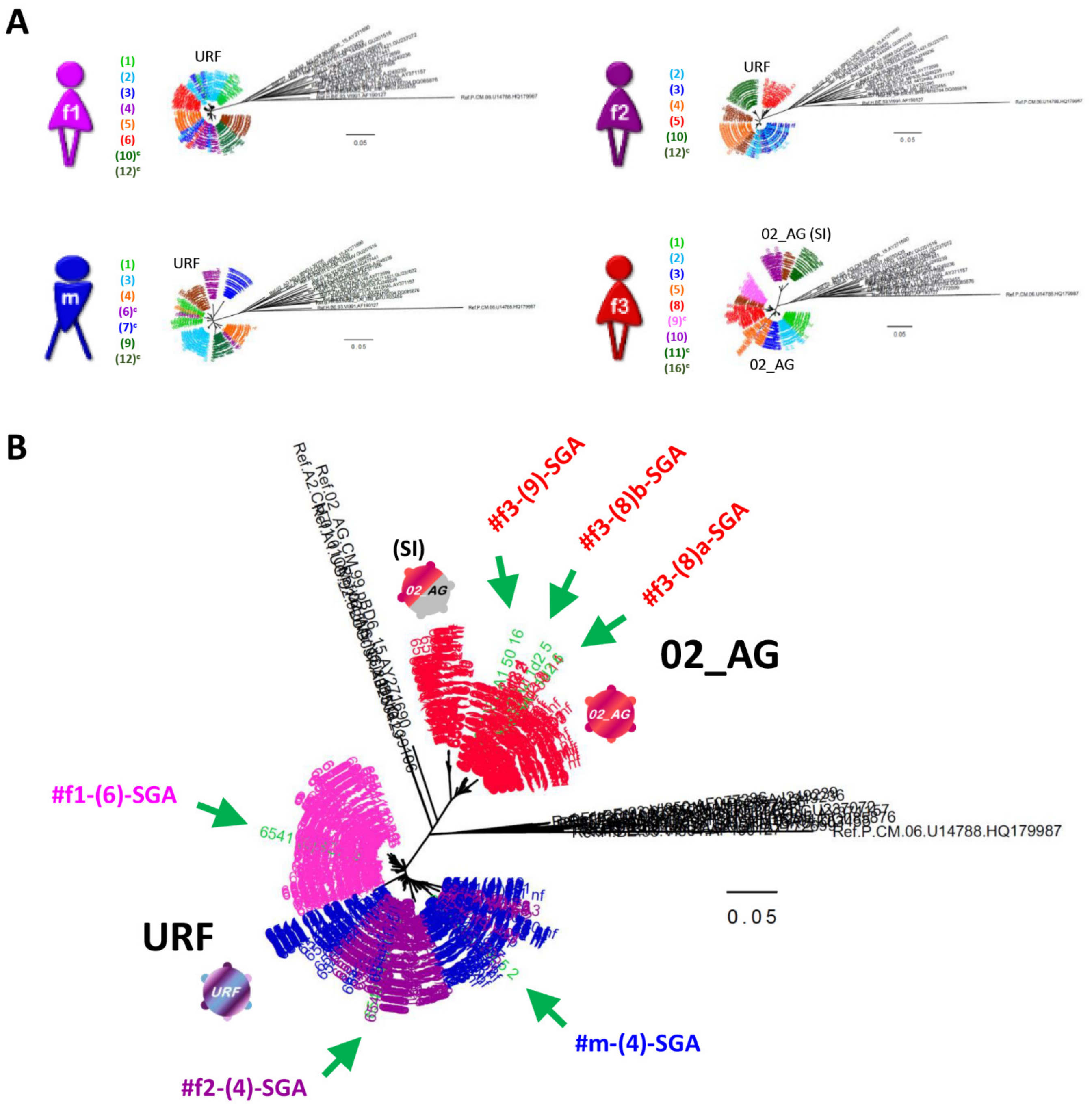

Supplementary Figure 2 | Longitudinal analysis of participants' *env* sequences in Neighbor-joining phylogenetic trees. (**A**) Neighbor-joining trees (Kimura two-parameter substitution model) displaying *env* sequences covering HIV region 6225–7817 according to HXB2 numbering. Separate trees are shown for each individual and the taxa are color-coded according to time point as indicated in the legend to the left of each tree. At least 20 sequences were analyzed per time point, generated using single genome amplification (SGA) or cloning. Participant #f3's CRF02_AG sequences are sub-classified according to their relatedness to sequences prior or post superinfection (SI), the latter labeled SI. Most sequences are derived from plasma viral RNA and a few from cellular (PBMC) proviral DNA (highlighted with c in superscript after the time point). (**B**) Combined neighbor-joining tree (Kimura two-parameter model) displaying the four participants' *env* sequences (HIV region 6225-7817 according to HXB2 numbering) color-coded according to study participant. Six SGA *env* sequences that were selected for detailed genetic and functional analyses are highlighted in green, *i.e.*, three representatives of the bulk of unique recombinant form (URF) sequences from #f1, #f2, and #m, as well as three representatives of the bulk of CRF02_AG sequences from #f3 before initiation of antiretroviral treatment and occurrence of superinfection. The bar indicates a genetic distance of 5%. Reference sequences from the Los Alamos Database are shown in black. PBMC: peripheral blood mononuclear cells.

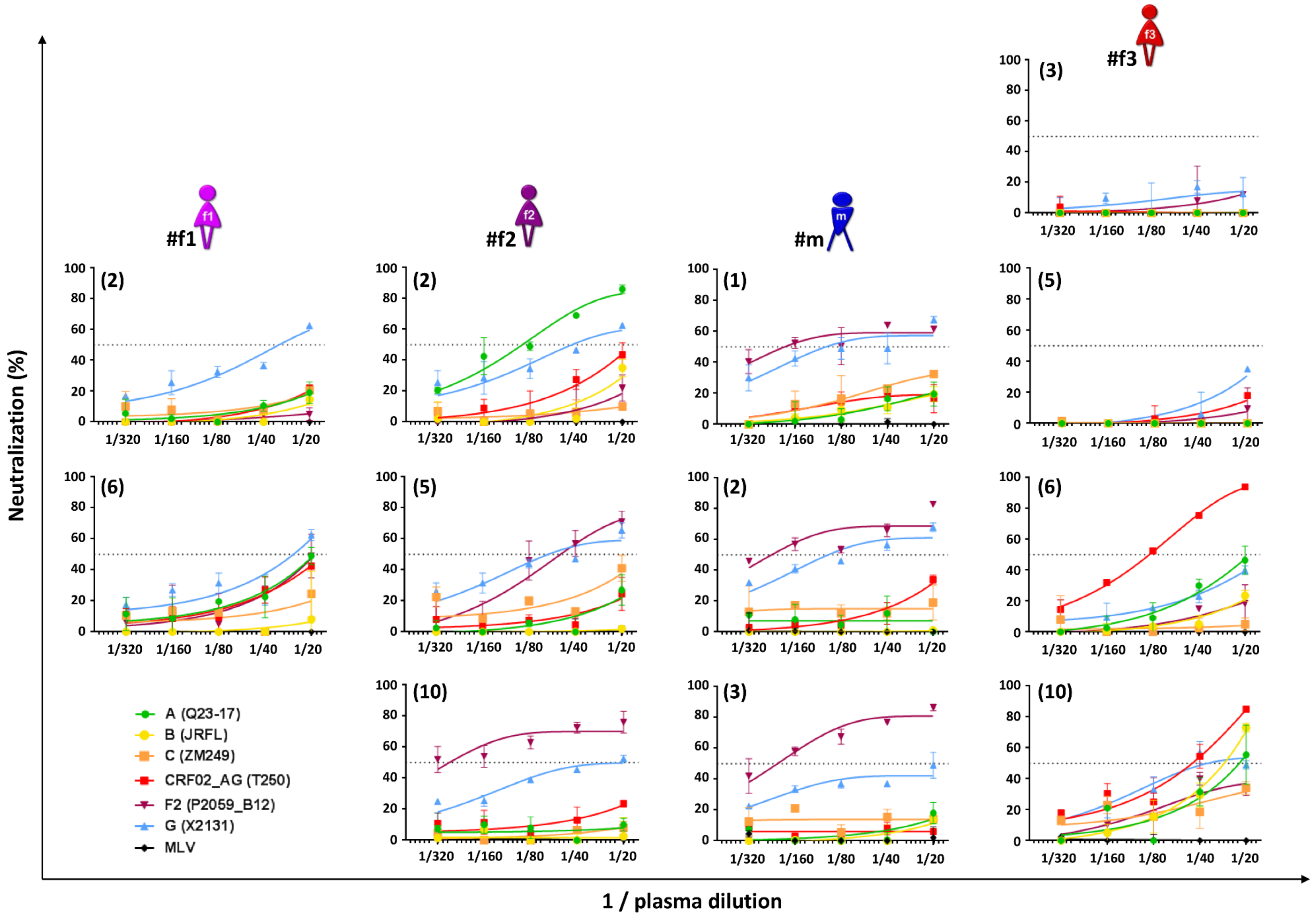

Supplementary Figure 3 | Heterologous neutralization responses in four Cameroonian HIV-1-infected individuals. Neutralization curves from plasma samples for #f1, #f2, #m, and #f3, shown as nonlinear regression fits of percent neutralization at the reciprocal plasma dilution. Longitudinal

plasma samples (time points in parentheses) were tested in 2-fold serial dilutions from 1/20 to 1/320 against pseudoviruses of diverse subtypes as indicated in the figure legend (HIV strains in parentheses). Virus pseudotyped with Env of murine leukemia virus (MLV) was used as negative control. Experiments were done at least twice, of which one representative experiment is displayed. The dashed line marks 50% neutralization.

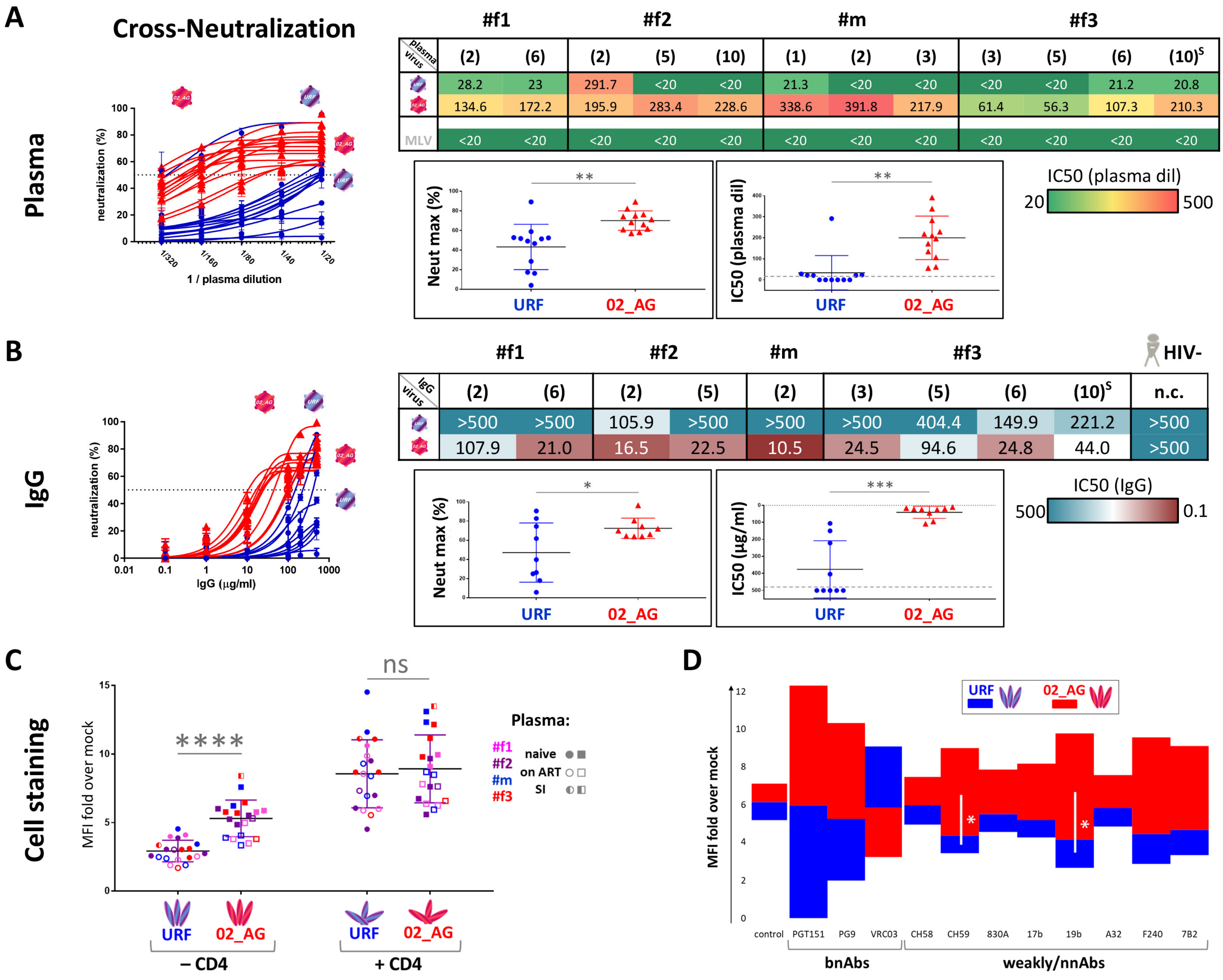
A
Cross-Neutralization
Plasma
neutralization (%)
1 / plasma dilution
1/320 1/160 1/80 1/40 1/20
#f1 #f2 #m #f3
plasma virus
(2) (6) (2) (5) (10) (1) (2) (3) (3) (5) (6) (10)$
28.2 23 291.7 <20 <20 21.3 <20 <20 <20 <20 21.2 20.8
134.6 172.2 195.9 283.4 228.6 338.6 391.8 217.9 61.4 56.3 107.3 210.3
MLV <20 <20 <20 <20 <20 <20 <20 <20 <20 <20 <20 <20
Neut max (%)
IC50 (plasma dil)
URF 02_AG
**
IC50 (plasma dil)
20 500
B
IgG
neutralization (%)
IgG (μg/ml)
0.01 0.1 1 10 100 1000
#f1 #f2 #m #f3 HIV-
IgG virus
(2) (6) (2) (5) (2) (3) (5) (6) (10)$ n.c.
>500 >500 105.9 >500 >500 >500 404.4 149.9 221.2 >500
107.9 21.0 16.5 22.5 10.5 24.5 94.6 24.8 44.0 >500
Neut max (%)
IC50 (μg/ml)
URF 02_AG
*
***
IC50 (IgG)
500 0.1
C
Cell staining
MFI fold over mock
ns
****
Plasma:
#f1 #f2 #m #f3
naive
on ART
SI
URF 02_AG URF 02_AG
– CD4 + CD4
D
MFI fold over mock
URF 02_AG
control PGT151 PG9 VRC03 CH58 CH59 830A 17b 19b A32 F240 7B2
bnAbs
weakly/nnAbs

Supplementary Figure 4 | Quantitative analysis of autologous plasma and IgG neutralization and cell-associated binding. (**A** and **B**) Autologous/cross-neutralization responses of longitudinal plasma (**A**) and plasma-purified IgG samples (**B**) are illustrated as heat maps (right) according to the color-coded IC_{50} scheme of plasma dilutions (dil) or IgG concentrations (µg/mL), respectively. Time points of plasma samples are indicated in parentheses; S in superscript indicates the time point when superinfection was detected in #f3. Two autologous viruses were studied pseudotyped with Env of study participants #m and #f3 before initiation of antiretroviral treatment and occurrence of superinfection (#m-(4)-SGA and #f3-(8)a-SGA, see **Figure 3**). Murine leukemia virus (MLV) and IgG purified from a healthy, HIV negative Cameroonian individual were used as negative controls (n.c.). Plasma and IgG dilutions needed for 50% neutralization of the corresponding pseudovirus (IC_{50}) were calculated using nonlinear regression fits of neutralization curves (left). Undetectable IC_{50} values are indicated as <20 (plasma) or >500 (IgG). The entirety of plasma and IgG cross-neutralization curves, color-coded according to the virus are shown on the left. Neutralization curves were done with 2-fold serial plasma dilutions from 1/20 to 1/320 or IgG concentrations of 500, 200, 100, 10, 1, and 0.1 µg/mL. The dashed line marks 50% neutralization. Differences in mean neutralization sensitivities of viruses against participants' IgG samples are indicated by the location of the schematic viruses in relation to the X-axis (IC_{50}) and Y-axis (maximum neutralization). Neut max and IC_{50} values were quantitatively compared between viruses in the boxed plots using unpaired t-tests (two-tailed) and all statistically significant results displayed. Mean and standard deviation are indicated. Color-coding was done according to pseudovirus. Undetectable IC_{50} values are shown below the dashed line (at plasma dilution 0 or IgG concentration 500ug/mL). (**C**) Cell-surface staining of 293T cells co-transfected with viral *env* (URF and CRF02_AG) isolated from study participants #m or #f3 (as described above) and an empty (-CD4) or human CD4 expressor (+CD4). Staining of native Env was done using longitudinal plasma samples as indicated. The data points are color-coded according to the participant; additionally, the filled circles correspond to ART-naïve time points, open circles to time points where the participants received ART and bi-colored diamonds to the time points when superinfection was detected in #f3. Means and standard deviation of two experiments are displayed. Cell staining against URF and CRF02_AG Env was statistically compared using unpaired two-tailed t-tests. (**D**) Cell-surface staining of 293T cells transfected with *env* of participants' autologous viruses (as above). Staining of native Env was done using a panel of monoclonal antibodies directed against the gp120-gp41 interface (PGT151), V2q (PG9), CD4bs (VRC03), V2p (CH58, CH59), V2i (830A), CD4i (17b, A32), V3 (19b) and gp41 (F240, 7B2), grouped according to broadly or weakly/non-neutralizing antibodies (bnAbs, weakly/nnAbs). Means of three experiments are shown in stacked bar graphs, sorted in descending order (highest values on top). All statistically significant differences between Envs per mAb are indicated (unpaired two-tailed t-test); * $P<0.05$; ** $P<0.005$; *** $P<0.0005$; **** $P<0.0001$; URF: unique recombinant form.

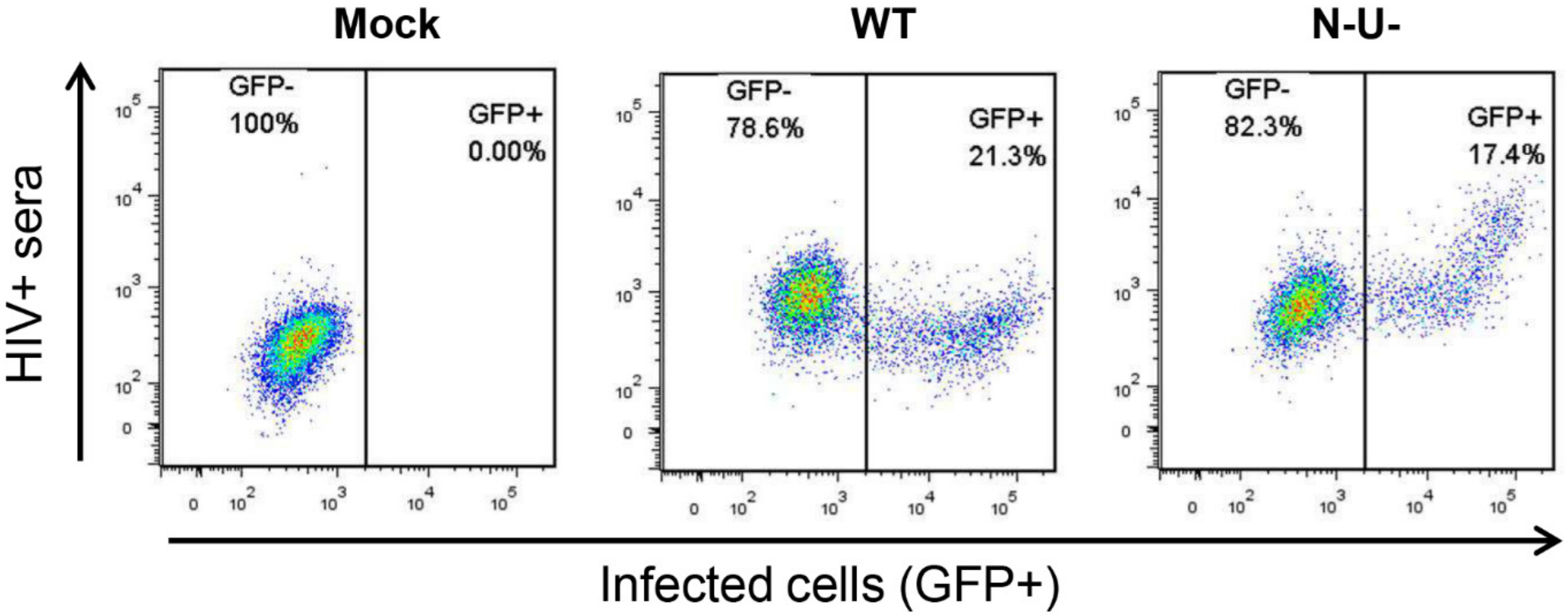

Supplementary Figure 5 | Detection of infected cells by flow cytometry. CEM.NKR cells infected with wildtype (pNL4.3-ADA-GFP) or Nef- and Vpu-deficient (pNL4.3-ADA-GFP/N-U-) viruses were stained with 1:1000 diluted HIV+ sera followed by second antibody (goat anti-human Ab coupled to Alexa Fluor 647). Infected cells were identified as being GFP+ by flow cytometry (BD LSR II).

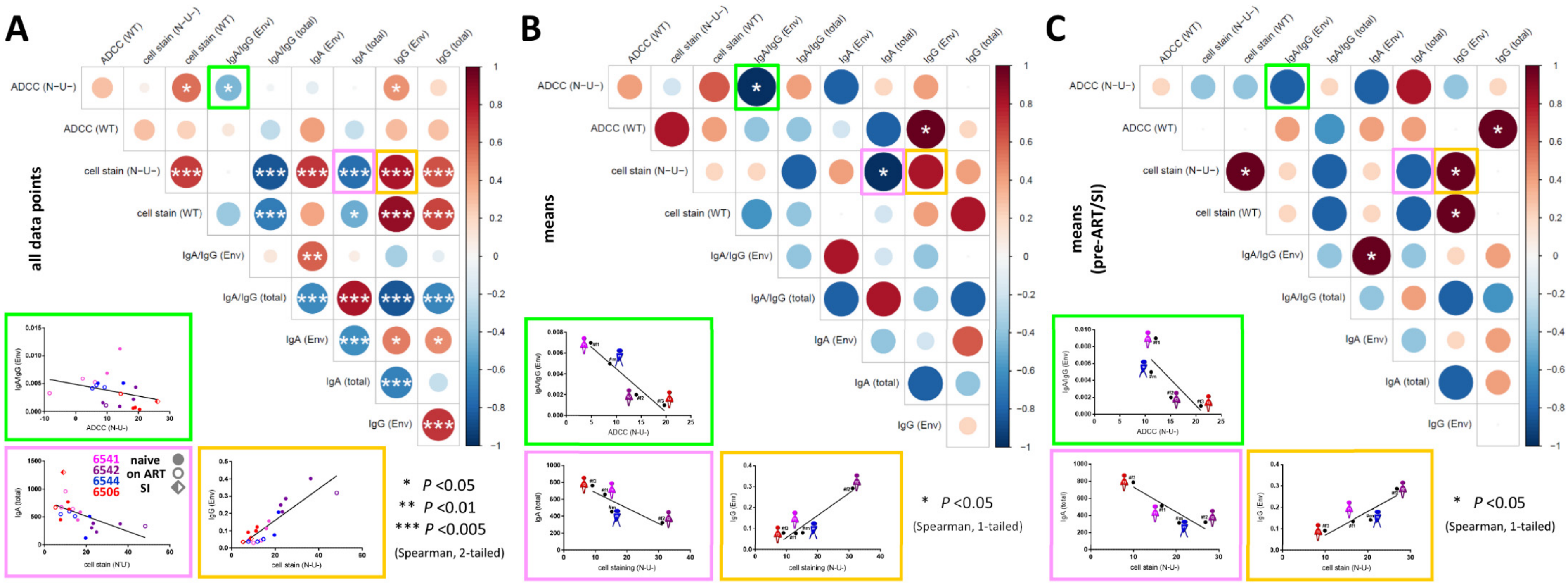

Supplementary Figure 6 | Correlation analysis of antibody-dependent cellular cytotoxicity (ADCC), cell-associated Env binding, IgA and IgG levels. Correlation analysis was performed on three data sets, *i.e.*, all data points (**A**), means per individual and parameter (**B**), and means from time points pre-ART and pre-superinfection (**C**). A correlogram is shown for each data set with circles, sized and color-coded according to linear regression coefficients (*r*) of correlations between the indicated parameters. Asterisks indicate all statistically significant correlations according to the provided *P*-value scheme. Selected linear regression analyses are shown at the bottom, boxed in the same colors as in the correlogram above. The data points in the boxed plots for (**A**) are color-coded according to the individual; additionally, in (**A**), the filled circles correspond to ART-naïve time points, open circles to time points where the participants received ART, and bi-colored diamonds to time points when superinfection was detected in #f3. ART: antiretroviral treatment; cell stain: cell-associated Env binding; Env: Env-specific; N-U-: infection experiment with Nef- and Vpu-deficient virus; WT: wild-type virus infection.

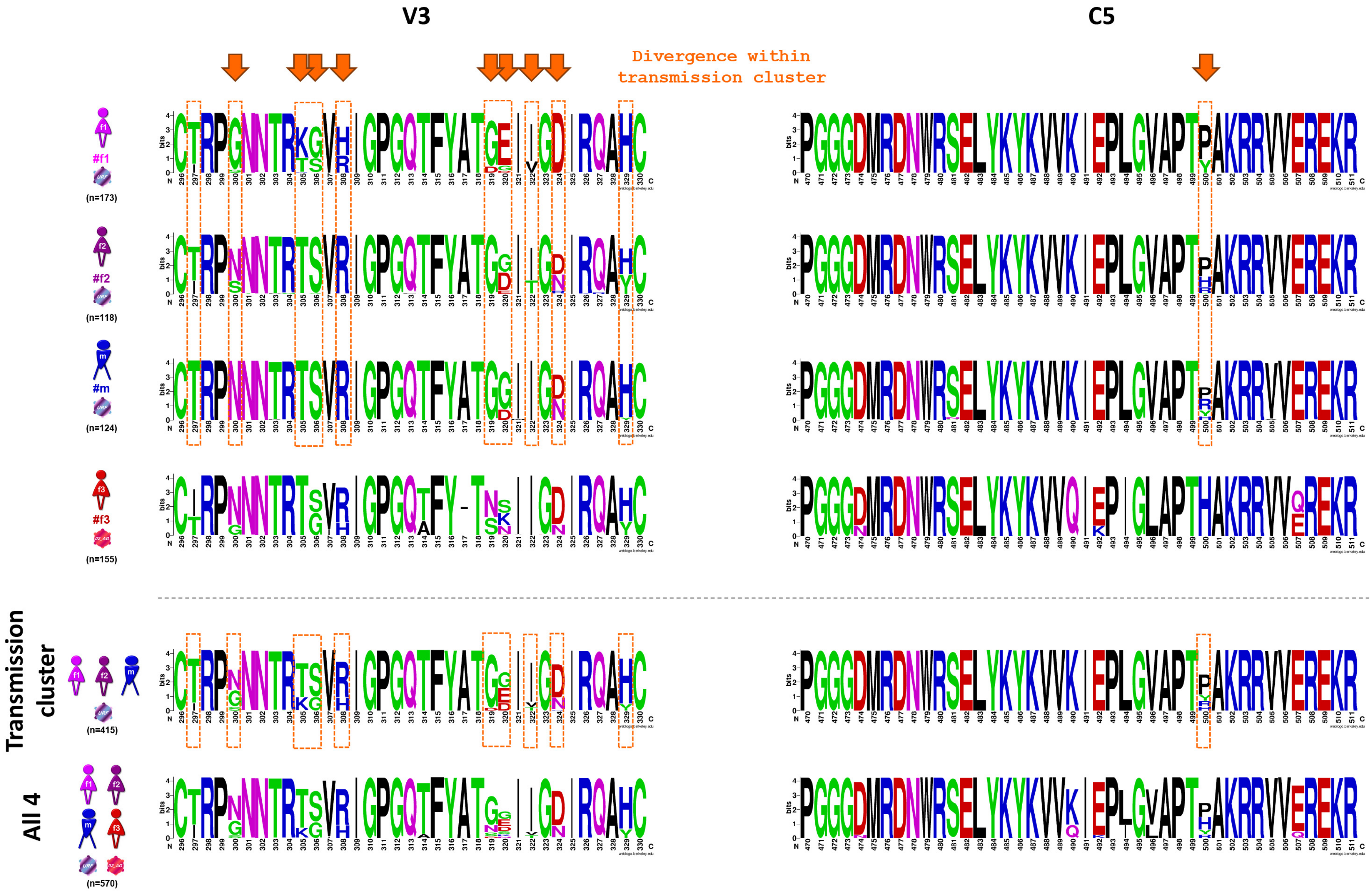

V3
C5
Divergence within transmission cluster
#f1
(n=173)
#f2
(n=118)
#m
(n=124)
#f3
(n=155)
Transmission cluster
(n=415)
All 4
(n=570)

Supplementary Figure 7 | Sequence analysis of viral envelope V3 and C5 regions. Sequence logo analysis of the variable V3 and constant C5 region (aa 296-330/1 and aa 470-511 according to HXB2 Env numbering, respectively) using all functional, longitudinal Env sequences determined in the four study participants. Sequence logos were created to assess the conservation of each position of the sequence motifs. The analyses were done for each participant separately (top) as well as for the three individuals of the transmission cluster (415 sequences in total) and for all four study participants together (570 sequences in total) (bottom). Sites of divergence in viral sequences of the transmission cluster are boxed in orange and highlighted with arrows on top; aa: amino acids.

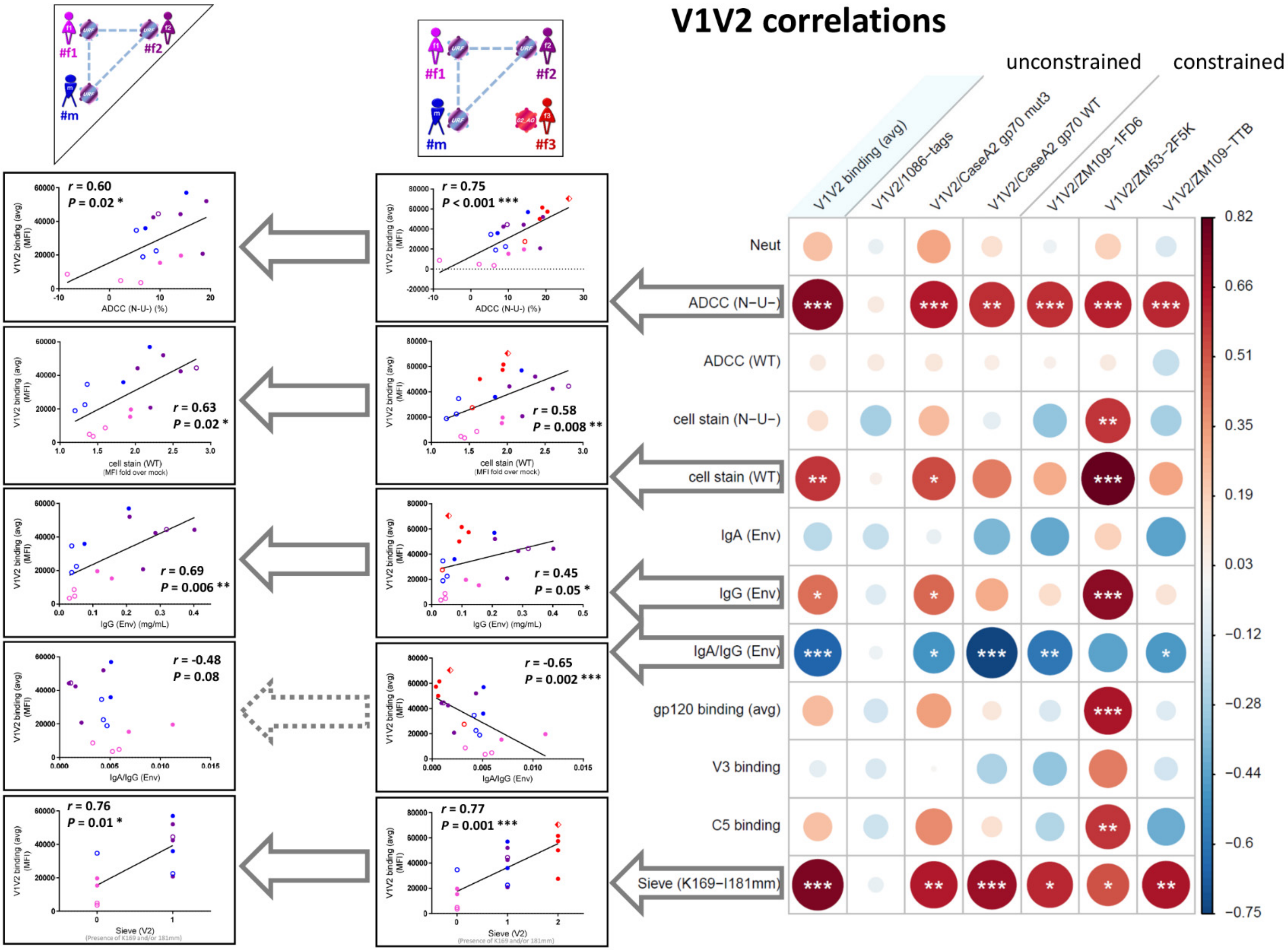
V1V2 correlations
unconstrained
constrained
#f1
#f2
#m
#f3
V1V2 binding (avg)
V1V2/1086-tags
V1V2/CaseA2 gp70 mut3
V1V2/CaseA2 gp70 WT
V1V2/ZM109-1FD6
V1V2/ZM53-2F5K
V1V2/ZM109-TTB
Neut
ADCC (N-U-)
ADCC (WT)
cell stain (N-U-)
cell stain (WT)
IgA (Env)
IgG (Env)
IgA/IgG (Env)
gp120 binding (avg)
V3 binding
C5 binding
Sieve (K169-I181mm)
0.82
0.66
0.51
0.35
0.19
0.03
−0.12
−0.28
−0.44
−0.6
−0.75
r = 0.60
P = 0.02 *
r = 0.75
P < 0.001 ***
ADCC (N-U-) (%)
r = 0.63
P = 0.02 *
r = 0.58
P = 0.008 **
cell stain (WT)
(MFI fold over mock)
r = 0.69
P = 0.006 **
r = 0.45
P = 0.05 *
IgG (Env) (mg/mL)
r = -0.48
P = 0.08
r = -0.65
P = 0.002 ***
IgA/IgG (Env)
r = 0.76
P = 0.01 *
r = 0.77
P = 0.001 ***
Sieve (V2)
(Presence of K169 and/or 181mm)
V1V2 binding (avg) (MFI)

Supplementary Figure 8 | Correlation analysis of the V1V2 binding response. Correlation analysis between V1V2 binding levels (six scaffolded V1V2 proteins and the average thereof) and selected immune responses from **Figures 4**, **5**, and **7**, and the presence of residue K169 and mismatch (mm) at I181 (according to the sieve analysis in the RV144 vaccine trial) in contemporaneous viral sequences. Analyses were performed using all data points from the four study participants (middle and right) or the three epidemiologically linked individuals (left) according to **Supplementary Table 2**. The correlations are summarized in a correlogram with circles, sized and color-coded according to linear regression coefficients (*r*) between the indicated parameters (red for positive correlation; blue for inverse correlation). All statistically significant correlations are indicated by asterisks (two-tailed Spearman rank, * $P<0.05$; ** $P<0.01$; *** $P<0.005$). All significant linear regression analyses with V1V2 (avg) using the four-participant data set are depicted as scatter plots in the middle. Corresponding analyses were done using the data set of the three epidemiologically-linked participants (on the left). The data points are color-coded according to the individual; additionally, the filled circles correspond to antiretroviral treatment (ART)-naïve time points, open circles to time points at which participants received ART, and bi-colored diamonds to time points when superinfection was detected in #f3. Linear regression fits are shown for all correlations that reach statistical significance and are highlighted by a bold arrow. Sieve (V2) on the x-axis is scaled according to the presence of K169 (score of 1), mismatch at I181 (score of 1), or both (score of 2) in contemporaneous viruses of participants. Env: Env-specific; WT: wild-type infection; N-U-: Nef- and Vpu- deficient virus infection; avg: average.

A

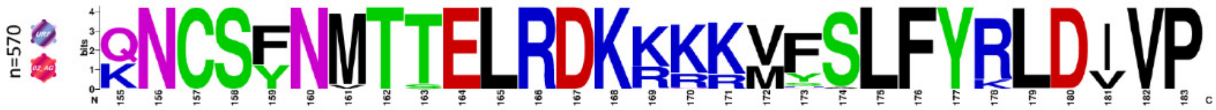

	155	156	157	158	159	160	161	162	163	164	165	166	167	168	169	170	171	172	173	174	175	176	177	178	179	180	181	182	183
conserved		*	*	*		*		*	*	*	*	*	*	*							*	*	*	*	*	*		*	*
Major residue	Q	N	C	S	F	N	M	T	T	E	L	R	D	K	K	K	K	V	F	S	L	F	Y	R	L	D	I	V	P
Minor residues	K				Y		V		(S)						R E	R Q	R	M	Y S N H D	A				(K)			V		

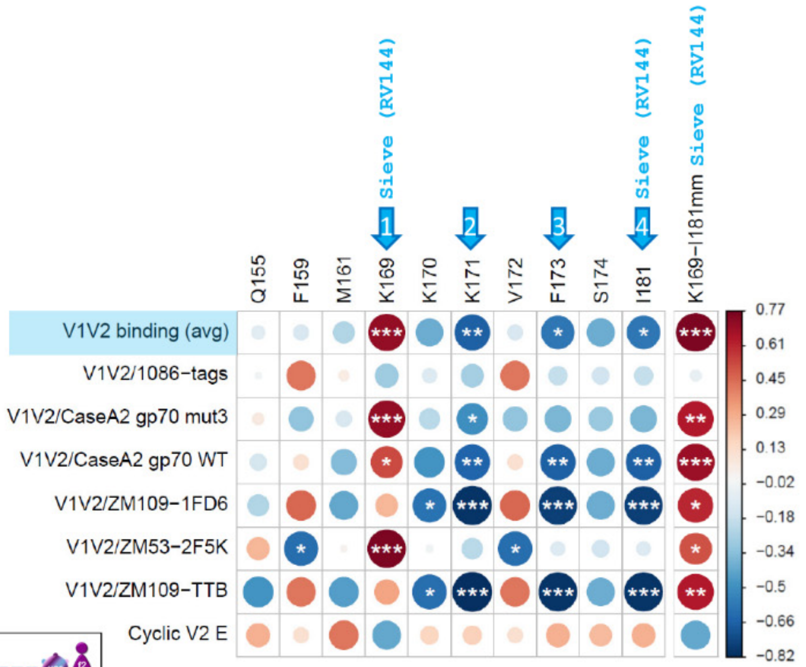

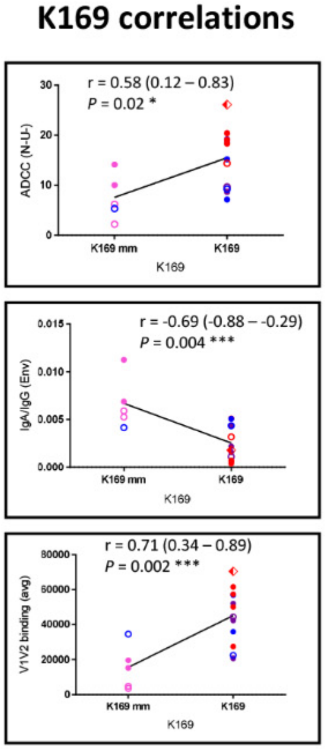

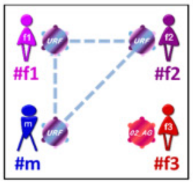

site scanning	V1V2 binding (avg)				
	r	*(95% CI)*	*P*	adjusted *P*	
				BH	Bonferroni
Q155	-0.10	(-0.56 – 0.42)	0.74	0.74	1
F159	-0.12	(-0.58 – 0.39)	0.67	0.73	1
M161	-0.25	(-0.66 – 0.28)	0.37	0.49	1
1 K169	0.71	(0.34 – 0.89)	0.002	0.009	0.03
K170	-0.40	(-0.74 – 0.12)	0.13	0.20	1
2 K171	-0.67	(-0.87 – -0.26)	0.005	0.02	0.06
V172	-0.12	(-0.58 – 0.39)	0.67	0.73	1
3 F173	-0.58	(-0.83 – -0.12)	0.02	0.04	0.23
S174	-0.41	(-0.75 – 0.11)	0.12	0.20	1
4 I181	-0.58	(-0.83 – -0.12)	0.02	0.04	0.23
K169-I181mm (Sieve RV144)	0.77	(0.44 – 0.91)	<0.001	0.004	0.007

K169

B

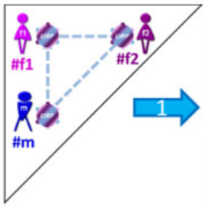

site scanning	V1V2 binding (avg)				
	r	*(95% CI)*	*P*	adjusted *P*	
				BH	Bonferroni
Q155	0.59	(0 – 0.87)	0.06	0.07	0.45
F159	-0.76	(-0.93 – -0.31)	0.01	0.01	0.07
M161	na	na	na	na	na
1 K169	0.76	(0.31 – 0.93)	0.01	0.01	0.07
K170	na	na	na	na	na
K171	-0.48	(-0.83 – 0.15)	0.17	0.17	1
V172	-0.76	(-0.93 – -0.31)	0.01	0.01	0.07
F173	na	na	na	na	na
S174	na	na	na	na	na
I181	na	na	na	na	na
K169-I181mm (Sieve RV144)	0.76	(0.31 – 0.93)	0.01	0.01	0.07

Supplementary Figure 9 | Site-scanning analysis of the immunodominant V1V2 region and correlation analysis with V1V2 binding. (**A**) Top: Sequence logo analysis of the immunodominant V1V2 region (aa 155-183 according to HXB2 numbering) using all functional, longitudinal Env sequences determined in the four study participants. The table below differentiates variable amino acids from conserved amino acids (indicated by an asterisk; N156 and N160 boxed in orange; T163 and R178 are <100% conserved, but 100% conserved for the data points for which immune responses were determined [gray asterisk]). For the variable amino acids, both major and minor residues are indicated as present in the dataset. If the presence of the major residue was found to be associated with increased or decreased V1V2 binding (according to the correlation analysis in the middle section), the major residues are highlighted with dark red or blue background, respectively. Middle: All non-conserved sites were subjected to a non-parametric correlation analysis (Spearman rank, two-tailed) with the binding responses against six scaffolded V1V2 proteins, the average (avg) of the latter, and a V2 cyclic peptide (see **Figure 7A**). The entirety of linear correlations for the individual sites and the combined epitope is shown in a correlogram using circles, sized and color-coded according to linear regression coefficients (*r*). All correlations that reach statistical significance are indicated by stars (Spearman rank, * $P<0.05$; ** $P<0.01$; *** $P<0.005$). Sites that reach statistically significant correlations with V1V2 binding (avg) are highlighted in light blue/with light blue arrows in the correlogram and tables. Identified V1V2 correlation sites that correspond to the sites of V1V2 selection pressure, as observed in the RV144 vaccine trial, are indicated. The multi-site analysis included the presence of K169 and a mismatch (mm) at 181 (according to the Sieve analysis in RV144). Bottom: Correction for multiple comparisons. Table summarizing correlations (Spearman rank) between longitudinal V1V2 binding (avg) and the presence of the indicated amino acid residue(s) in the contemporaneous viruses. Correlation coefficients *r*, 95% confidence intervals (CI), significance *P* and multiple comparison-adjusted *P*-values (Benjamini Hochberg and Bonferroni method) are shown for each correlation; statistically significant results ($P<0.05$) are highlighted in light blue. Right: For site K169, which achieved significant *P*-values for both BH and Bonferroni corrections, linear regression analyses against selected immune responses are displayed, *i.e.*, K169 against V1V2 binding (avg), against Env-specific IgA/IgG ratios, and against antibody-dependent cellular cytotoxicity (ADCC) of cells infected with Nef- and Vpu-deficient (N-U-) virus (statistically significant according to the provided *r*- and *P*-values; 95% CI shown in brackets). The individual data points are color-coded according to the participant; additionally, the filled circles correspond to antiretroviral treatment (ART)-naïve time points, open circles to time points where the participants received ART, and bi-colored diamonds to time points when superinfection was detected in #f3. (**B**) V1V2 site scanning and correlation analysis were similarly done for the data set on the three epidemiologically-linked individuals, as described above for the four participants (**A**). The results are summarized in a table displaying two-tailed Spearman rank correlations between longitudinal V1V2 binding (avg) and the presence of the indicated amino acid residue(s) in the contemporaneous viruses (see above). Sites that remain significantly associated with V1V2 binding (avg) in the three linked participant data set are highlighted with a blue arrow; aa: amino acids; avg: average; mm: mismatch; na: not applicable (due to 100% conserved sites in the given data set).

A

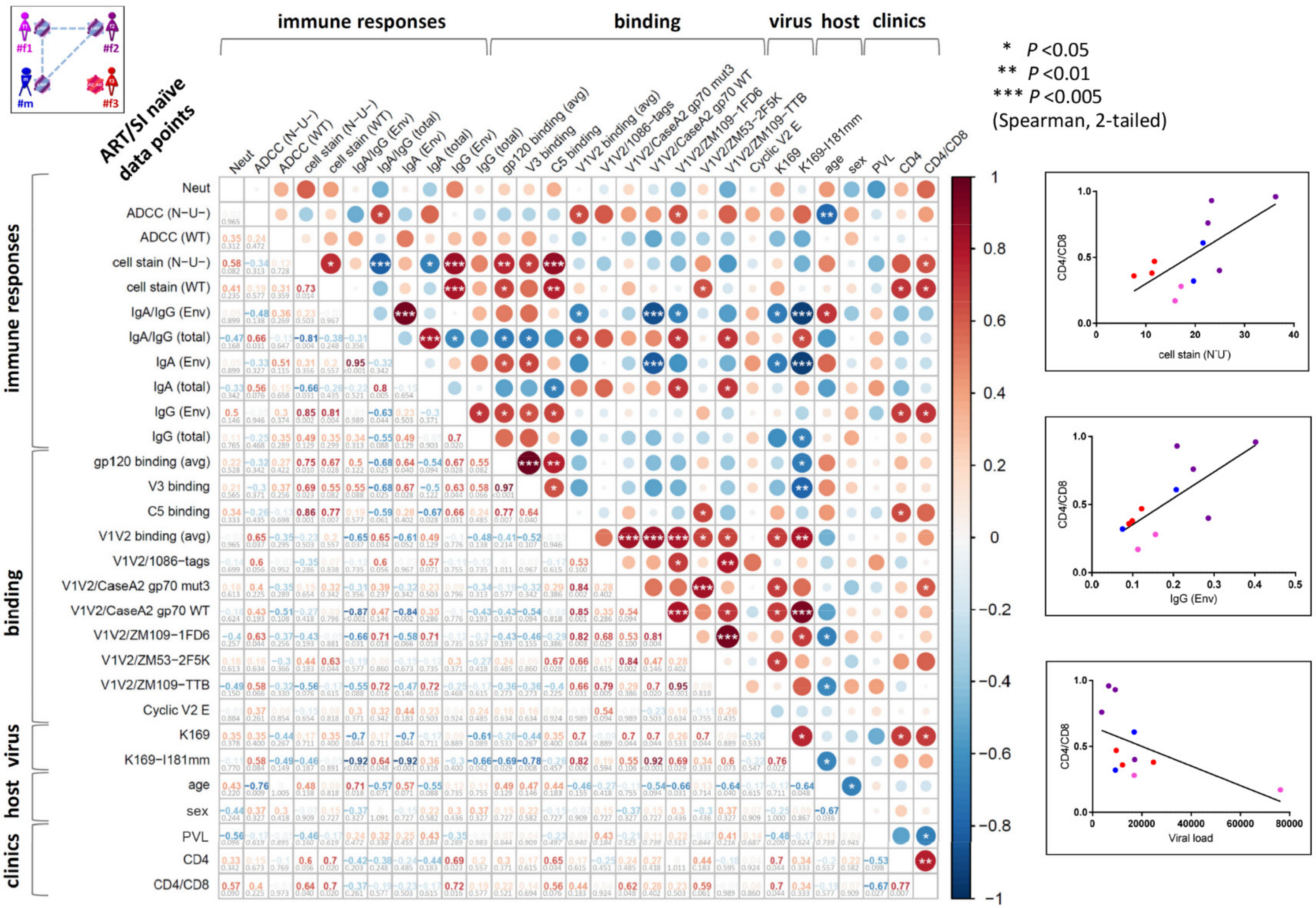

B

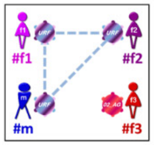

single parameter	CD4/CD8 r	(95% CI)	P	adjusted P BH	adjusted P Bonferroni
Neut	0.66	(0.20 – 0.88)	0.05	0.12	0.45
ADCC (N-U-)	0.40	(0.13 – 0.62)	0.22	0.28	0.90
ADCC (WT)	-0.01	(-0.02 – 0)	0.97	0.97	1.00
cell stain (N-U-)	0.64	(0.23 – 0.86)	0.04	0.07	0.34
cell stain (WT)	0.70	(0.26 – 0.90)	0.02	0.05	0.24
IgA (Env)	-0.23	(-0.38 – -0.07)	0.50	0.54	1.00
IgG (Env)	0.72	(0.27 – 0.91)	0.02	0.05	0.21
IgA/IgG (Env)	-0.37	(-0.58 – -0.12)	0.26	0.30	0.90
V1V2/CaseA2 mut3	0.62	(0.22 – 0.84)	0.05	0.07	0.34
K169	0.70	(0.17 – 0.92)	0.04	0.07	0.34
PVL	-0.67	(-0.88 – -0.24)	0.03	0.06	0.27

multiple parameter	CD4/CD8 r	(95% CI)	P	adjusted P BH	adjusted P Bonferroni
IgG (Env) & cell stain (N-U-)	0.72	(0.27 – 0.91)	<0.001	<0.001	<0.001
IgG (Env) & cell stain (WT)	0.75	(0.29 – 0.93)	<0.001	<0.001	<0.001
V1V2/CaseA2 mut3 & K169	0.71	(0.27 – 0.91)	<0.001	<0.001	0.001
ADCC (N-U-) & IgA/IgG (Env)	0.45	(0.15 – 0.68)	0.02	0.05	0.24
ADCC (WT) & IgA/IgG (Env)	0.40	(0.13 – 0.62)	0.04	0.07	0.34

C

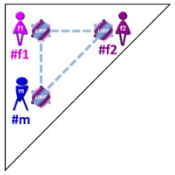

single parameter	CD4/CD8 r	(95% CI)	P	adjusted P BH	adjusted P Bonferroni
Neut	0.61	(0.09 – 0.87)	0.17	0.21	1.00
ADCC (N-U-)	0.48	(0.06 – 0.75)	0.24	0.26	1.00
ADCC (WT)	-0.29	(-0.51 – -0.04)	0.50	0.50	1.00
cell stain (N-U-)	0.86	(0.16 – 0.98)	0.01	0.03	0.17
cell stain (WT)	0.52	(0.07 – 0.79)	0.20	0.22	1.00
IgA (Env)	-0.60	(-0.86 – -0.09)	0.13	0.18	1.00
IgG (Env)	0.76	(0.12 – 0.95)	0.04	0.07	0.59
IgA/IgG (Env)	-0.79	(-0.96 – -0.13)	0.03	0.06	0.45
V1V2/CaseA2 mut3	0.69	(0.10 – 0.92)	0.07	0.10	1.00
K169	0.79	(0.13 – 0.96)	0.07	0.10	1.00
PVL	-0.78	(-0.96 – -0.13)	0.03	0.06	0.47

multiple parameter	CD4/CD8 r	(95% CI)	P	adjusted P BH	adjusted P Bonferroni
IgG (Env) & cell stain (N-U-)	0.86	(0.16 – 0.98)	<0.001	<0.001	<0.001
IgG (Env) & cell stain (WT)	0.76	(0.12 – 0.95)	<0.001	<0.001	<0.001
V1V2/CaseA2 mut3 & K169	0.78	(0.13 – 0.96)	<0.001	<0.001	<0.001
ADCC (N-U-) & IgA/IgG (Env)	0.93	(0.20 – 0.97)	<0.001	<0.001	<0.001
ADCC (WT) & IgA/IgG (Env)	0.79	(0.13 – 0.96)	<0.001	<0.001	<0.001

Supplementary Figure 10 | Correlation analysis including the full matrix of study parameters using data from time points pre-ART and pre-superinfection. (**A**) Correlogram using circles, sized and color-coded according to linear regression coefficients (*r*) from correlations between the indicated parameters (based on **Supplementary Table 2**) and grouped according to clinical, host, viral, binding (Luminex) or immunological parameters. Asterisks indicate all statistically significant correlations according to the provided *P*-value scheme in the upper right corner. In the lower left triangle of the correlogram**,** *r*-values are shown in the same color-code as applied in the correlogram; *P*-values are shown below in gray. To the right of the correlogram, linear regression analyses are shown for three selected parameters (X-axis) that exhibited statistically significant correlations with CD4/CD8 ratios (Y-axis), both in data sets comprising four and three study participants (see below, **B** and **C**). The individual data points are color-coded according to participant (#f1 pink, #f2 purple, #m blue, and #f3 red); * $P<0.05$; ** $P<0.01$; *** $P<0.005$. (**B** and **C**) CD4/CD8 correlation analysis using data points of the four study participants (**B**) or the subset of three epidemiologically-linked individuals (**C**)**.** The tables summarize linear and multivariate correlation analyses (two-tailed Spearman rank) among various viral, immunologic, and clinical parameters and CD4/CD8 ratios using data points pre-ART and pre-superinfection. Correlation coefficients *r*, 95% confidence intervals (CI), significance *P* and multiple-comparisons-adjusted *P*-values (Benjamini-Hochberg and Bonferroni method) are displayed for each correlation; statistically significant results ($P<0.05$) are highlighted in green. ADCC: antibody-dependent cellular cytotoxicity; ART: antiretroviral treatment; avg: average; Env: Env-specific; Neut: Neutralization breadth-potency score; N-U-: infection experiment with Nef-, Vpu-deficient virus; PVL: plasma viral load; WT: wild-type virus; ADCC: Antibody-dependent cellular cytotoxicity.

A

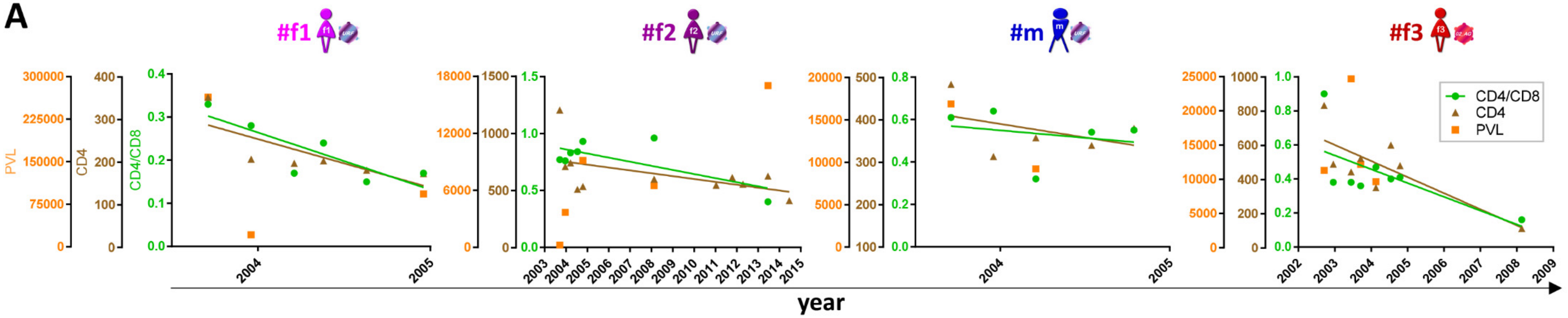

	CD4/CD8	CD4 [cells/mm³]
N	6	6
R^2	0.69	0.60
**Slope (progression/year)**	**-0.13**	**-112.6**

	CD4/CD8	CD4 [cells/mm³]
N	7	11
R^2	0.48	0.27
**Slope (progression/year)**	**-0.04**	**-25.21**

	CD4/CD8	CD4 [cells/mm³]
N	5	5
R^2	0.05	0.17
**Slope (progression/year)**	**-0.07**	**-79.4**

	CD4/CD8	CD4 [cells/mm³]
N	8	8
R^2	0.44	0.62
**Slope (progression/year)**	**-0.08**	**-94.6**

B

	Time point	Time post EDI	Time post diagnosis	ART
#f1	earliest: (2)	<2 years	1 year 3 months	naive
	2nd earliest: (6)	< 3 years	2 years 3 months	naive
#f2	earliest: (2)	< 1 year	10 months	naive
	2nd earliest: (5)	< 2 years	1 year 8 months	naive
#m	earliest: (1)	< 3 years	7 months	naive
	2nd earliest: (3)	< 3.5 years	1 year 2 months	naive
#f3	earliest: (3)	< 1.5 years	9 months	naive
	2nd earliest: (5)	< 2 years	1 year	naive

C

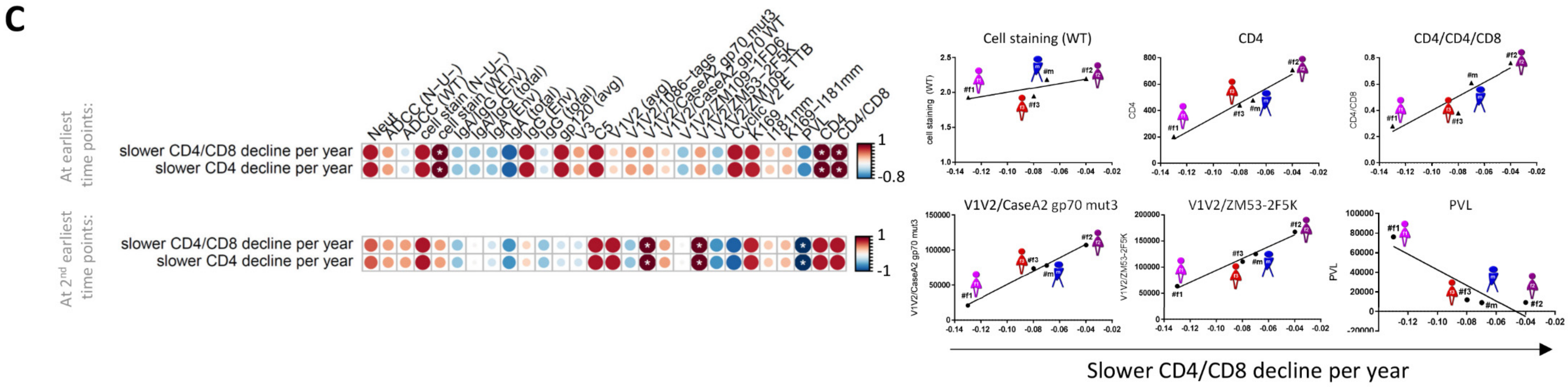

Supplementary Figure 11 | Early predictors of disease progression. (**A**) Clinical progression of four HIV-1 infected participants during ART-naïve years of infection. Dot plots show for each participant longitudinal values of CD4/CD8 ratios, CD4 counts (cells/mm^3), and plasma viral load (PVL [cps/ml]). Linear regression fits are indicated for CD4/CD8 and CD4 counts with statistics shown in the tables below including the number of data points (N), the goodness of fit (R^2), and slope of linear regression (progression per year). (**B**) Table summarizing the characteristics of the early/intermediate study time points of the four participants used for the determination of predictors of disease progression. Estimated dates of infection (EDI) are based on BEAST analyses (**Figure 1, Supplementary Table 1**). (**C**) Correlogram using circles, sized and color-coded according to linear regression coefficients (*r*) from correlations between the indicated parameters (red for positive correlation; blue for inverse correlation). All statistically significant correlations are indicated by an asterisk (one-tailed Spearman rank, * $P<0.05$; n=4). Two early/intermediate time points were studied as indicated. The parameters on the x-axis are based on **Supplementary Table 2**. The parameters on the y-axis are the rates of disease progression as determined in (**A**) using slopes (decline) of CD4/CD8 ratios and absolute CD4 counts per year. The correlation analysis was done for the two most early study time points for which the full set of functional and structural data were generated (as shown in **B**). To the right of the correlogram, linear regression analyses are shown for all statistically significant correlations (shown for the CD4/CD8 data set only). The data points are labeled according to the participant. ADCC: antibody-dependent cellular cytotoxicity; ART: antiretroviral treatment; avg: average; BEAST: Bayesian evolutionary analysis by sampling trees; EDI: estimated date of infection; Env: Env-specific; mm: mismatch; Neut: Neutralization breadth-potency score; N-U-: infection experiment with Nef-, Vpu-deficient virus; PVL: plasma viral load; WT: wild-type virus; ADCC: Antibody-dependent cellular cytotoxicity.

Supplementary Tables

Supplementary Table 1 | Parameter estimates of the discrete phylogeography models.

Bayes factor test support for discrete diffusion rates[a]			
From	To	Bayes Factor	Support
#f1	#f2	<1.0	None
#f1	#m	<3.0	None
#f2	#f1	<2.0	None
#f2	#m	>500.0	Very strong
#m	#f1	>4.0	Strong
#m	#f2	>500.0	Very strong

Markov Jumps[b]	Mean Magnitude	95% HPD Interval	Effective Sample size
#f1 to #f2	0.042	0 – 0.19	3685
#f1 to #m	0.623	0 – 2.09	1575
#f2 to #f1	0.136	0 – 1.06	2559
#f2 to #m	4.804	3.96 – 7.30	2683
#m to #f1	0.636	0 – 1.53	1178
#m to #f2	5.549	4.04 – 6.96	3534
Total Count	11.8	9.29 – 14.38	1680

Markov Rewards[c]	Mean	95% HPD Interval	Effective Sample Size
#f1	46.02	32.5 – 62.2	3294
#f2	38.11	31.0 – 47.6	341
#m	61.66	38.17 – 87.4	3181

Subject	Root Probability[d]
#m	0.52
#f1	0.40
#f2	0.08

Subject	Estimated date of Infection[e]	95% HPD Interval	Incidence Testing
#m	2001	1999 – 2003	< April 2003
#f1	2002	2001 – 2003	< April 2003
#f2	2003	2003 – 2004	< April 2003
#f3	2002	2002 – 2003	April – Sept 2002

[a] Bayes factor test values for significant historic diffusion-dynamics links among individuals. [b] Quantification of the magnitude of the inferred historic diffusion events using Markov jumps. [c] Estimates of the time spent in each host using Markov rewards and inferred using stochastic mapping techniques. [d] Root probabilities. [e] Comparison of estimated dates of infection based on time to the most recent common ancestor (TMRCA), 95% higher posterior density interval (95% HPD) of TMRCA, and incidence testing (**Figure 1**).

Supplementary Table 2 | Combined data matrix used in correlation analyses.

a	immune responses											binding											virus						host		clinics			HLA
	Neut	ADCC (N^-U^-)	ADCC (WT)	cell stain (N^-U^-)	cell stain (WT)	IgA/IgG (Env)	IgA/IgG (total)	IgA (Env)	IgA (total)	IgG (Env)	IgG (total)	gp120 binding (avg)	V3 binding	C5 binding	V1V2 binding (avg)	V1V2/ 1086-tags	V1V2/ CaseA2 gp70 mut3	V1V2/ CaseA2 gp70 WT	V1V2/ ZM109-1FD6	V1V2/ ZM53-2F5K	V1V2/ ZM109-TTB	Cyclic V2 E	K169	K171mm	F173mm	I181mm	K169-I181mm (Sieve RV144)	K169-K171mm-F173mm-I181mm	age	sex (f=1; m=0)	PVL	CD4	CD4/CD8	Prot. HLA
#f1-(2)	0.24	10.00	3.40	17.14	1.93	0.00687	0.122	1.018	583.83	0.156	4.960	156743.8	170610.0	8563.8	15306.4	818.3	27105.5	1417.5	5627.3	52450.8	4419.0	3730.0	0.0	1.0	1.0	1.0	0.0	0.0	30.8	1	16847	202	0.28	0
#f1-(6)	0.20	14.14	2.13	15.93	1.94	0.01124	0.132	1.155	444.85	0.113	3.821	183058.0	185591.8	16178.5	19619.0	4742.3	20852.5	1772.0	11273.3	63786.5	15287.5	11044.5	0.0	1.0	1.0	1.0	0.0	0.0	31.8	1	76242	169	0.17	0
#f1-(7)[T]	na	-8.34	0.12	13.55	1.60	0.00329	0.199	0.134	643.50	0.044	3.500	103681.8	133373.5	11841.3	8673.3	4349.8	5851.5	925.8	8919.3	18044.0	13949.8	9642.0	na	na	na	na	na	na	34.9	1	0	360	0.38	0
#f1-(10)[T]	na	2.22	-4.40	8.07	1.39	0.00591	0.279	0.270	671.31	0.046	2.565	76020.8	96325.3	6020.5	4841.5	2228.0	2480.5	330.5	4501.3	11070.0	8438.5	5057.5	0.0	1.0	1.0	1.0	0.0	0.0	40.3	1	0	789	0.65	0
#f1-(12)[T]	na	6.20	-5.42	10.02	1.44	0.00525	0.358	0.143	957.94	0.030	2.845	71701.9	90728.8	4906.8	3555.6	1607.5	2518.5	454.8	3344.3	8780.0	4628.8	4144.8	0.0	1.0	1.0	1.0	0.0	0.0	42.9	1	0	621	0.74	0
#f2-(2)	4.48	18.45	3.19	22.59	2.20	0.00218	0.080	0.489	304.59	0.249	4.106	168058.9	170730.0	131917.0	20796.6	1079.0	38402.0	4480.0	4445.3	72125.5	4248.0	6117.8	1.0	1.0	1.0	1.0	1.0	1.0	23.6	1	3686	709	0.76	0
#f2-(5)	0.55	19.18	2.67	23.33	2.37	0.00435	0.139	0.830	382.65	0.209	3.032	180558.9	176888.8	169644.0	52000.6	2154.5	107430.8	13689.0	11535.8	167385.8	9807.8	5269.3	1.0	1.0	1.0	1.0	1.0	1.0	24.4	1	9158	534	0.93	0
#f2-(6)	na	14.06	-2.19	36.31	2.03	0.00093	0.074	0.335	376.22	0.402	5.411	178594.5	176849.0	167512.0	44268.7	1239.3	87232.8	25773.0	12526.8	125015.8	13824.5	4023.5	na	na	na	na	na	na	27.8	1	6500	599	0.96	0
#f2-(10)	0.86	8.67	1.86	24.92	2.60	0.00158	0.070	0.432	229.43	0.286	3.643	189654.4	184689.5	181009.0	42438.6	1119.8	62004.5	32433.3	7730.8	143075.8	8267.8	3070.0	1.0	1.0	1.0	1.0	1.0	1.0	33.1	1	17036	626	0.40	0
#f2-(12)[T]	na	9.68	-1.99	48.27	2.81	0.00111	0.079	0.311	334.76	0.319	4.465	186141.5	179187.0	177554.0	44450.4	1516.3	61126.8	35586.5	7479.3	152673.3	8320.5	3555.8	1.0	1.0	1.0	1.0	1.0	1.0	35.8	1	0	686	0.86	0
#m-(1)	2.30	15.20	0.86	21.59	2.19	0.00510	0.161	0.970	510.85	0.207	3.398	164481.4	159226.0	136243.0	56979.4	2402.3	134085.0	20403.8	11585.3	162765.5	10634.8	7179.8	1.0	0.5	1.0	1.0	1.0	1.5	37.9	0	16847	479	0.61	0
#m-(3)	1.17	7.12	-1.57	19.67	1.84	0.00507	0.106	0.351	119.53	0.075	1.210	145874.4	128955.0	118785.0	35942.9	734.0	78369.8	5207.5	3391.8	125015.5	2938.8	2246.5	1.0	1.0	1.0	1.0	1.0	1.0	38.4	0	9204	321	0.32	0
#m-(6)[T]	na	9.24	1.69	14.62	1.33	0.00435	0.322	0.185	512.26	0.051	1.666	121679.5	96362.0	75359.0	22469.7	557.5	57375.3	1809.5	2010.5	71092.0	1973.5	3458.8	1.0	1.0	1.0	1.0	1.0	1.0	42.3	0	0	289	0.58	0
#m-(10)[T]	na	6.61	-0.13	11.89	1.20	0.00471	0.312	0.148	595.75	0.037	2.079	104681.5	77421.8	47558.8	18919.8	1234.5	47084.0	1281.8	2512.0	57856.5	3549.8	3828.5	na	na	na	na	na	na	47.7	0	0	921	0.77	0
#m-(12)[T]	na	5.33	2.35	7.70	1.36	0.00416	0.292	0.149	548.66	0.037	2.061	78236.0	32038.5	53999.5	34664.0	67808.3	59326.8	47610.8	35468.8	-3400.5	1169.8	76256.3	0.0	1.0	1.0	1.0	0.0	0.0	50.3	0	0	856	1.84	0
#f3-(3)	0	19.03	-0.61	11.23	1.95	0.00072	0.250	0.070	768.91	0.099	3.086	104583.4	44605.3	12277.5	61539.4	2369.5	90072.8	50079.0	52250.0	129323.5	45141.8	4310.3	1.0	0.0	0.0	0.0	2.0	4.0	19.3	1	24696	441	0.38	1
#f3-(5)	0	18.35	-2.99	7.60	1.64	0.00060	0.233	0.053	449.13	0.091	1.924	85654.4	36718.5	10471.0	50109.5	1564.3	74105.5	39786.0	40560.0	110614.3	34027.3	5558.3	1.0	0.0	0.0	0.0	2.0	4.0	19.5	1	12132	520	0.36	1
#f3-(6)	1.50	20.42	2.67	11.74	1.94	0.00038	0.221	0.046	638.78	0.122	2.887	98958.1	46777.8	7312.8	57340.3	2414.0	83030.0	46788.8	46536.3	124847.3	40425.8	3359.3	1.0	0.0	0.0	0.0	2.0	4.0	19.9	1	9597	349	0.47	1
#f3-(10)[S]	11.44	26.12	-0.30	8.98	2.01	0.00179	0.388	0.100	1302.25	0.056	3.403	131513.8	93327.5	3553.8	70432.0	2338.3	91196.0	65407.5	72716.3	128958.5	61975.5	3186.3	1.0	0.0	0.0	0.0	2.0	4.0	28.3	1	58544	50	0.02	1
#f3-(16)[T]	na	14.40	-0.97	5.25	1.54	0.00318	0.410	0.096	669.64	0.035	1.634	54940.6	18088.5	2401.3	27507.9	903.8	41863.8	24942.5	21377.0	59291.5	16668.8	2352.5	1.0	0.0	0.0	0.0	2.0	4.0	32.9	1	0	879	1.48	1

b	Neut	ADCC (N^-U^-)	ADCC (WT)	cell stain (N^-U^-)	cell stain (WT)	IgA/IgG (Env)	IgA/IgG (total)	IgA (Env)	IgA (total)	IgG (Env)	IgG (total)	gp120 binding (avg)	V3 binding	C5 binding	V1V2 binding (avg)	V1V2/ 1086-tags	V1V2/ CaseA2 gp70 mut3	V1V2/ CaseA2 gp70 WT	V1V2/ ZM109-1FD6	V1V2/ ZM53-2F5K	V1V2/ ZM109-TTB	Cyclic V2 E	K169	K171mm	F173mm	I181mm	K169-I181mm (Sieve RV144)	K169-K171mm-F173mm-I181mm	age	sex (f=1; m=0)	PVL	CD4	CD4/CD8	Prot. HLA
#f1	0.22	12.07	2.76	16.54	1.93	0.00905	0.127	1.087	514.34	0.134	4.390	169900.9	178100.9	12371.1	17462.7	2780.3	23979.0	1594.8	8450.3	58118.6	9853.3	7387.3	0.0	1.0	1.0	1.0	0.0	0.0	31.3	1	46545	186	0.23	0
#f2	1.96	15.09	1.38	26.78	2.30	0.00226	0.091	0.521	323.22	0.287	4.048	179216.7	177289.3	162520.5	39876.1	1398.1	73767.5	19093.8	9059.6	126900.7	9037.0	4620.1	1.0	1.0	1.0	1.0	1.0	1.0	27.2	1	9095	617	0.76	0
#m	1.74	11.16	-0.36	20.63	2.02	0.00508	0.134	0.661	315.19	0.141	2.304	155177.9	144090.5	127514.0	46461.2	1568.1	106227.4	12805.7	7488.5	143890.5	6786.8	4713.1	1.0	0.8	1.0	1.0	1.0	1.3	38.2	0	13026	400	0.47	0
#f3	0.50	19.27	-0.31	10.19	1.84	0.00056	0.235	0.056	618.94	0.104	2.632	96398.6	42700.5	10020.4	56329.8	2115.9	82402.8	45551.3	46448.8	121595.0	39864.9	4409.3	1.0	0.0	0.0	0.0	2.0	4.0	19.6	1	15475	437	0.40	1

Data acquired from the results of this study and clinical data assessment. [a] Longitudinal data points for the four participants. Viral signatures from #f3-(6) (dark green) are derived from viruses collected five months later (see **Figure 1**; time point 8). HLA typing was performed for at least two longitudinal time points per participant (black) and consistent values were imputed for the remaining time points per participant (gray). Eight immunological, clinical, and viral parameters that were selected for subsequent multivariate/network analyses (**Figure 10A**) are highlighted in green. The values for V1V2 and gp120 binding are averaged (avg) antibody binding levels to six different constrained and unconstrained V1V2 proteins or two different gp120 proteins, respectively (**Figure 7A**). The values for viral signatures mirror the majority of viral sequences per participant and time point. [b] Means per participant and parameter from time points pre-ART and pre-superinfection. The value for the parameter age is set to the date October 2004; all participants at this age were at a stage of chronic HIV-1 infection, before the initiation of ART, and before the occurrence of superinfection. All other means are calculated from applicable data points ([a]). ART: antiretroviral treatment; Env: Env-specific; na: not available; Neut: Neutralization breadth-potency score; N-U-: infection experiment with Nef-, Vpu-deficient virus; VL: viral load; WT: wild-type virus.
